# Hypertension-Linked Mutation of α-Adducin Increases CFTR Surface Expression and Activity in HEK and Cultured Rat Distal Convoluted Tubule Cells

**DOI:** 10.1371/journal.pone.0052014

**Published:** 2012-12-21

**Authors:** Anna Mondini, Francesca Sassone, Davide Antonio Civello, Maria Lisa Garavaglia, Claudia Bazzini, Simona Rodighiero, Valeria Vezzoli, Fabio Conti, Lucia Torielli, Giovanbattista Capasso, Markus Paulmichl, Giuliano Meyer

**Affiliations:** 1 Department of Life Sciences, Università degli Studi di Milano, Milano, Italy; 2 Fondazione Filarete, Milano, Italy; 3 Prassis Research Institute, Sigma Tau, Settimo M.se, Italy; 4 Department of Internal Medicine, Second University of Napoli, Napoli, Italy; 5 Institut of Pharmacology and Toxicology, Paracelsus Medical University, Salzburg, Austria; Cinvestav-IPN, Mexico

## Abstract

The CFTR (Cystic Fibrosis Transmembrane Conductance Regulator) activity and localization are influenced by the cytoskeleton, in particular by actin and its polymerization state. In this study we investigated whether the expression of the hypertensive mutations of α-adducin (G460W-S586C in humans, F316Y in rats), an actin capping protein, led to a functional modification of CFTR activity and surface expression. The experiments were performed on HEK293 T cells cotransfected with CFTR and the human wild type (WT) or G460W mutated α-adducin. In whole-cell patch-clamp experiments, both the CFTR chloride current and the slope of current activation after forskolin addition were significantly higher in HEK cells overexpressing the G460W adducin. A higher plasma membrane density of active CFTR channels was confirmed by cell-attached patch-clamp experiments, both in HEK cells and in cultured primary DCT cells, isolated from MHS (Milan Hypertensive Strain, a Wistar rat (Rattus norvegicus) hypertensive model carrying the F316Y adducin mutation), compared to MNS (Milan Normotensive Strain) rats. Western blot experiments demonstrated an increase of the plasma membrane CFTR protein expression, with a modification of the channel glycosylation state, in the presence of the mutated adducin. A higher retention of CFTR protein in the plasma membrane was confirmed both by FRAP (Fluorescence Recovery After Photobleaching) and photoactivation experiments. The present data indicate that in HEK cells and in isolated DCT cells the presence of the G460W-S586C hypertensive variant of adducin increases CFTR channel activity, possibly by altering its membrane turnover and inducing a retention of the channel in the plasmamembrane. Since CFTR is known to modulate the activity of many others transport systems, the increased surface expression of the channel could have consequences on the whole network of transport in kidney cells.

## Introduction

Adducins (α, β, and γ) are cytoskeletal F-actin end-capping proteins that play a role in restricting actin filament length [1], by binding directly to F-actin and bundling actin filaments [2]. Furthermore they promote the binding of spectrin to actin [3], [4], thus regulating the assembly of the subcortical cytoskeletal membrane network [5]. In epithelial cells, adducin is associated with the spectrin-based membrane skeleton and spectrin-adducin-ankyrin complexes link membranes to the actin cytoskeleton [6]. In this cell type adducins are expecially abundant at cell-cell contact sites [7] and are also present in the clathrin-coated vesicle (CCV) compartment, associated with clathrin [8].

Both in a cell free system and in a kidney cell line, α-adducin mutations (F316Y and G460W-S586C in rats and humans respectively) interfere with actin assembly and polymerization, leading to a higher final level of filamentous actin [9]. These adducin mutations have also been found to be significantly related to Na^+^-sensitive hypertension development by influencing cation and anion transport mechanisms in renal epithelia [10]–[13].

In renal epithelial cells, the transfection with mutated (F316Y) rat α-adducin induces an increase in Na^+^/K^+^ pump activity [9]. This upregulation is in part depending on a decrease of pump endocytosis from the basolateral membrane [8], likely because of increased adaptor protein 2 (AP-2) phosphorylation [14]. Furthermore in vitro, mutated adducin variants have been shown to directly activate the Na^+^/K^+^-ATPase, by accelerating the rate of conformational change [13] and increasing Src-dependent Na^+^/K^+^-ATPase phosphorylation and activity [15].

Na^+^/K^+^-ATPase is not the only renal transport system affected by mutations of the adducin gene. In Thick Ascending Limb (TAL) and Distal Convoluted Tubule (DCT) cells of Milan Hypertensive rats (MHS), a model system of Na^+^-sensitive hypertension bearing the F316Y adducin mutation [16], an increase of the activity and/or expression of NKCC2 [17], [18], NCC (Na^+^-Cl^−^ cotransporter), and of ClC-K chloride channels has been observed [11], suggesting that adducin mutations can directly or indirectly impact on several Na^+^ and Cl^−^ transport systems, especially in the distal segments of the nephron.

CFTR is one of the chloride channels expressed in the kidney. Its expression and functional activity has been demonstrated in several nephron tracts such as DCT, cortical collecting duct (CCT) and inner medullary collecting duct [19]. Despite the broad distribution, the role of CFTR in the kidney remains uncertain as there is no major disruption of renal function in cystic fibrosis patients [20], apart from a reduced renal excretion of NaCl. Nevertheless, *CFTR* is also a regulatory protein influencing the activity and localization of other membrane proteins as well and other renal Cl^−^ and Na^+^ channels and transporters could be influenced by CFTR [19], [21], [22], as observed in several cell types [23]. In proximal tubular cells, CFTR is present in intracellular vesicles along both the exocytic and the endocytic pathways, where by ensuring the chloride conductance dissipating the potential difference originated by the V-ATPase active H^+^-transport, it participates to endosomal acidification and therefore to receptor-mediated protein uptake by PT (proximal tubule) cells [24], [25].

CFTR channel activity and expression are regulated, besides other several intracellular factors like PKA, also by the cytoskeleton. Changes in actin filament organization modulate CFTR channel activity by a mechanism entailing a direct interaction between actin filaments and CFTR [26]. Furthermore CFTR intracellular dynamics and plasmamembrane expression are affected by cytoskeletal proteins. Efficient internalization of CFTR requires actin polymerization and association with the actin-binding motor protein Myosin VI [27], [28]. The surface localization of CFTR is stabilized also by other interacting proteins, such as the multidomain cytoskeletal protein filamin [29] and the PDZ-containing adaptor molecule NHERF1/EBP50, that connects CFTR to the microtubules network [30] and to the cortical actin cytoskeleton [31]. CFTR surface expression is also regulated through its trafficking and endocytotic recycling. It associates with SNARE proteins (syntaxin 1A, SNAP23) and endocytic adaptors such as AP-2, undergoing clathrin-mediated endocytosis [23], [32]. A balance between cytoskeletal tethering and capture by the endocytic machinery may be crucial to maintain a sufficient population of CFTR at the cell surface.

Since the hypertensive adducin variants influence the cytoskeleton and the AP-2 mediated endocytosis, we investigated whether CFTR could be influenced by these adducin mutations too. We performed electrophysiological, biochemical and fluorescence experiments to evaluate the influence of the hypertension-linked human G460W-S586C adducin variant (G460W adducin) on CFTR activity, expression and trafficking. These experiments demonstrated that this adducin mutation can actually modulate CFTR activity and surface expression in HEK 293 cells. To explore the physiological implications of adducin influence on CFTR channel activity, we performed additional experiments on Milan Hypertensive Rats (MHS) rats DCT primary cultured cells. Patch-clamp experiments demonstrated a higher channel activity in MHS rat cells compared to the corresponding normotensive strain (MNS), thus suggesting a possible role for adducin as a regulator of CFTR channel activity also *in vivo*.

## Materials and Methods

### cDNA Constructs

cDNA encoding human CFTR was subcloned from pcDNA3, kindly provided from M. Conese (Università degli Studi, Foggia), into the following expression vectors: pIRES2-EGFP (Clontech) for patch-clamp experiments, pEYFP-N and pEYFP-C (Clontech) for FRET and FRAP experiments, PAGFP for photoactivation experiments. cDNA encoding for human WT or G460W adducin was subcloned from pcDNA3.1, kindly provided by M.G. Tripodi (Prassis, Sigma Tau, Milan), into pECFP-C and pECFP-N (Clontech) for FRET, FRAP and photactivation experiments. In the case of FLAG coimmunoprecipitation experiments, a FLAG tag (sequence: DYKDDDDK) was inserted by mutagenesis (performed with QuikChange Site-Directed Mutagenesis Kit, Stratagene) at the C-t of adducin in the pcDNA3.1 vector, before the STOP codon. HA-immunopreciptation experiments were performed by using the pcDNA3.1-HA-adducin vector [8]. All cDNA constructs were confirmed by sequencing.

### Cell Culture and Transfection

#### Primary culture of DCT cells

Hypertensive male MHS Wistar rats (2 months old; body weight, 350 g) and corresponding control MNS were used. To obtain primary culture of DCT cells, DCT tubules were microdissected from the whole kidney according to a previously described method [11]. The experiments are in accordance with the Italian guidelines for laboratory animals (Protocol N° 5/2008). All surgery was performed under Avertin (2, 2, 2-Tribromoethanol) anesthesia, and all efforts were made to minimize suffering.

#### HEK cells

HEK293 cells stably transfected with WT (NU12 cells) or G460W (HU33 cells) α-adducin were kindly provided by Prassis, Sigma Tau, Milan [8]. Human embryonic kidney (HEK) 293T cells, NU12 and HU33 were cultured in MEM, 1% FBS and transiently transfected for FRET, Western blot or patch-clamp experiments as previously reported [33]. The maintenance of the expression of adducin was routinely assessed by Western blot and immunocytochemistry, as shown in Figure S1.

### Patch-clamp Experiments

Patch-clamp experiments were performed on NU12 (HEK cells stably transfected with the human wild-type adducin) and HU33 cells (expressing the G460W mutation) transiently transfected with a human CFTR expressing bicistronic vector (pIRES2-EGFP-CFTR), that allows to express both CFTR and EGFP (Enhanced Green Fluorescent Protein) as two separate proteins. The pIRES2-EGFP vector, expressing only EGFP, was used as control. Adducin and CFTR expression has been verified by Western blot. For whole-cell experiments the pipette solution contained (mM) 140 CsCl, 10 tetraethylammonium chloride, 0.5 EGTA, 2 MgCl_2_, 2 Mg–ATP, 5 glucose and 5 HEPES (pH 7.2, 320 mOsm) and the bath solution contained (mM) 145 NaCl, 4 CsCl,1 CaCl_2_, 1 MgCl_2_, 10 glucose and 10 HEPES (pH 7.4), 324 mOsm. All experiments were performed at room temperature (22–25°C). Pipettes were pulled from borosilicate glass and had resistances of 3–5 MΩ after fire polishing. Seal resistances were typically between 3 and 10 GΩ. After establishing the whole-cell configuration, CFTR was activated by adding 10 µmol/L forskolin. Currents were recorded using a EPC9 amplificator (HEKA, Germany) and low-pass filtered at 1 kHz. Mean currents were normalized as current densities (pA/pF). Cell capacitance was not statistically different in HU33 and NU12 cells (NU12: 17.7±1.3 pF, n = 14; HU33: 19.6±1.0 pF, n = 12). Forskolin activation time course analysis was performed after complete solution change in the chamber (within 30 s). The current amplitude was measured at a constant potential of +40 mV every 15 s until a steady state was obtained. The forskolin activation time course was fitted, using GraphPad Prism v5 software, with a sigmoidal function:
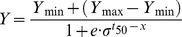



Where Y_min_ and Y_max_ are the minimum and the maximum Y values (pA/pF) respectively, σ is the rate constant for the rise of the sigmoidal function, and t_50_ is the time interval from the start to the inflection point of the sigmoidal function.

For cell-attached patch-clamp experiments the bath solution was an HBSS solution (Sigma, Italy), buffered at pH 7.4 with 10 mM Hepes, 304 mOsm. The micropipette solution (cell-attached and inside-out configurations) contained (mM): 115 N-Methyl Glucamine Cl, 30 TEACl, 2 CaCl_2_, 1 MgCl_2_, 10 HEPES (pH 7.4), 309 mOsm. All experiments were performed at room temperature (22–25°C). The patch pipette resistance was 5–10 MΩ, and seal resistance was 5–10 GΩ. Currents were recorded using a EPC9 amplificator (HEKA, Germany), digitized at 5 kHz and low-pass filtered at 0.2 kHz. The bath was earthed with an Ag/AgCl electrode immersed in the bath solution. Potential differences were expressed as overall potential differences, considering the junction potential, the holding potential, and the cell membrane potential, measured to be about -30 mV (current clamp experiments). Data analysis was performed by Bruxton Tac program. For single-channel studies of CFTR, voltage was stepped from −135 to +65 mV (overall potential) in 20 mV increments of 20 s duration. Slope conductance was calculated by dividing unitary current by the overall potential differences. The number of channels in a membrane patch was determined from the maximum number of simultaneous channel openings observed during the course of an experiment, as described [34]. For open probability (Po) analyses, lists of open and closed times were created using a half-amplitude crossing criterion for event detection. Transitions <3.32 ms in duration were excluded from the analyses, as filter risetime (tr = 0.3321/fc with fc = filter corner frequency) was 1.65 ms. As we usually observed more than one channel in a patch, the open probability of the single channel (Po) was calculated as previously reported [27]. Ramp I-V relationships were obtained by averaging currents generated by 10 ramps of voltage each of 2-s duration; holding voltage was 0 mV. All records were leakage corrected: basal currents with no active CFTR Cl^−^ channels were subtracted from those showing CFTR activity before averaging subtracted currents to generate the ensemble ramp I-V relationship of CFTR Cl^−^ currents [11].

### Western Blot

CFTR expression was assessed using the monoclonal anti-CFTR 24.1 antibody (R&D System) on lysates obtained from HEK293 clones stably expressing HA-tagged α-adducin WT (clone NU12) or G460W (clone HU33) and transiently expressing CFTR. Transfection efficiency has been verified by immunofluorescence and by the luciferase reporter methods (see supplemental material) and, with both methods, it was not significantly different between HU33 and NU12 cells (Figure S2). HU33 and NU12 cells transiently expressing CFTR were lysed in Triton lysis buffer (TLB: 1% Triton, 25 mmol/l Tris pH 7.4, 150 mmol/l NaCl) and protease inhibitors, (Complete EDTA-free Protease Inhibitor Cocktail (Roche). The lysate was spinned at 20000 g for 20 min and the supernatant saved. The isolation of the plasma membrane proteins was performed with the Plasma Membrane Protein Extraction Kit (MBL International Corporation), following manufacturer instructions. The reliability of plasma membrane separation has been verified by confirming the enrichment in the plasmamembrane fraction of cadherin, a plasmamembrane marker, compared to calreticulin, an endoplasmic reticulum marker (see Text S1 and Figure S2). Protein concentration in the samples was quantified by the Bradford assay (Biorad). All protein extracts were heated at 37°C for 20 minutes in SDS-PAGE solubilising buffer (57.85 mmol/l Tris HCl, 10% Glycerol, 2% SDS, 0.004% Bromophenol blue, pH 6.8) containing 7.5% Dithiothreitol, and microfuged for 1 minute. 100 µg of total proteins or 20 µg of membrane proteins were loaded in each lane and separated by SDS-PAGE-electrophoresis on a 6.5% polyacrylamide gel at 100 V for 2 hour. Following electrophoresis, proteins were transferred overnight onto a PVDF (Polyvinylidene Fluoride) membrane. After blocking in blocking buffer 1 (PBS pH 7.4, 2% semi-skimmed milk powder, 0.05% Tween-20) for 2 hours at room temperature, the membrane was incubated with the anti-CFTR 24.1 (R&D Systems) diluted 1∶1000 in the blocking buffer at 4°C overnight, followed, after several washing steps, by the secondary HRP-conjugated antibody (1∶10000) at room temperature for 1 hour. Detection was performed with the Immobilon ECL system (Millipore). In all other cases, after transfer, the membrane was exposed to blocking buffer 2 (PBS pH 7.4, 5% semi-skimmed milk powder, 0.1% Tween-20) for 1 hour at room temperature. In case of reprobing of the blot, the membrane was first exposed to a stripping solution (0.05 M glicine and 1% SDS) for 40 minutes at room temperature. The membrane was then incubated with the anti-cadherin (pan Cadherin antibody, Abcam, ab6529), anti-calreticulin (Calreticulin antibody, Abcam, ab4), anti-FLAG (FLAG M2 antibody, Sigma, F3165), anti-HA (HA antibody, Covance, Princeton, NJ) or anti-CFTR (CFTR H-182 antibody, Santa Cruz, sc-10747) antibody diluted in the blocking buffer at 4°C overnight. After several washing steps, membrane was exposed to secondary HRP-conjugated antibody at room temperature for 1 hour and washed before proceeding with the ECL detection.

At the end, the PVDF membrane was always stained with the amido black staining procedure in order to asses the efficiency of protein transfer and check for equal loading.

The densitometric analyses of the bands has been performed by means of the ImageJ software (NIH, USA).

### Immunofluorescence

Layers of DCT cells plated on glass coverslip were fixed with 3% paraformaldehyde and permeabilized with 0.1% Triton X-100. Non-specific binding was blocked with 5% BSA. Cells were then incubated at the presence of the mouse anti-CFTR (CFTR H-182 antibody, Santa Cruz, sc-10747) primary antibody at room temperature for 1 hour, followed by Cy2 anti-rabbit (1∶400 dilution, at room temperature for 1 hour) secondary antibody incubation (Jackson Labs). Images were acquired by a confocal microscope Leica TCS SP2 AOBS (Leica Microsystem, Heidelberg, Germany) provided with 63× Oil immersion objective (1.4 NA).

### FRAP

HEK293 T cells were transiently transfected (PEI method) with YFP-CFTR and CFP-adducin WT or CFP-adducin G460W. The experiments were performed on cells co-expressing both proteins, 48 h after-transfection, maintaining the cells at 37°C in PBS complemented with 4.5% glucose during the confocal observation. For imaging, YFP was excited with the 514 nm laser line and acquired between 525 and 600 nm, with a 63× (1.4 NA) oil immersion objective.

YFP-CFTR photobleaching and fluorescence recovery were analyzed in three different membrane ROIs. Fluorescence (F) recovery was followed for 150 sec.

At each time point the mean fluorescence in the ROI (F_ROI(t)_) was corrected for the background fluorescence (F_bg (t)_) and normalized for the mean fluorescence of a ROI that was not subjected to photobleching (F_noPB (t)_), subtracted of F_bg_, to correct for the photobleching of YFP due to the imaging procedure:
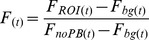



Finally, background and photobleaching-corrected F values were normalized for the F value measured just before photobleaching (F_prePB_), according to the equation:
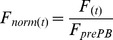



To calculate the mobile fraction (F_mob_%) and the half-time constant (t_1/2_), each ROI fluorescence (expressed as F_norm_) recovery was individually fitted with an exponential equation as follows:
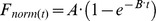
with A = F_norm_ _MAX_ represents the F value obtained by extrapolation at infinite time after recovery; B = 1/τ (1/s). τ is the first order rate constant for recovery and t_1/2_ (s) was calculated as 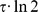; the mobile fraction (F_mob_%) was calculated according to the following equation:



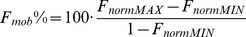
where F_norm MIN_ is the first F value after photobleaching (expressed as F_norm_).

For each experimental condition, all ROIs, t_1/2_ and F_mob_% values were averaged and the different populations were compared using Student’s t-test. All data derived from three different independent experiments.

### Photoactivation Experiments

HEK cells were transiently transfected (PEI method) with PAGFP-CFTR and CFP-adducin WT or CFP-adducin G460W. Cell were incubated in complete medium +5 µM Ikarugamycin (Ika cells) or complete medium +0.05% DMSO (the solvent of Ika, control cells) for 2 hours before the experiments. All the experiments were performed 48 h after-transfection at 37°C, maintaining the cells in the incubation medium+HEPES 10 mM during the confocal observation. For the imaging CFP and PAGFP where excited with the 458 nm and the 488 nm laser lines, respectively, and acquired in two different PMTs with the sequential scan mode. CFP emission bandwidth: 465–490 nm, PAGFP emission bandwidth: 500–550 nm. Pixel size: 80.2 nm, scan speed: 1400 Hz, 8 bit intensity resolution. Photoactivation was performed by exciting the PAGFP for 3 frames with the 405 nm laser line AOTF set to 50%, scan speed: 1400 Hz. The fluorescence decay in the photoactivation region (ROI) was followed for 150 s with a frame rate of 2 fr/s. Samples expressing CFP only were used to check for possible cross-excitation of CFP during the photoactivated PAGFP imaging with the 488 nm laser line: no cross-excitation were measured with the acquisition setting used. To check for possible photobleaching during the post photoactivation time-lapse, some cells where entirely photoactivated and the fluorescence were monitored for 150 s. No photobleaching was observed during the acquisition time. The images were analyzed with ImageJ and the obtained data with the Excel software (Microsoft). Briefly, after the photoactivation, the total fluorescence in the ROI (FROI_t*(i)*_) was corrected for the background (Fbg_t*(i)*_) and for the fluorescence before the photoactivation (FROI_t*(pre)*_−Fbg_t*(pre)*_). These corrected fluorescence values were normalized (F_norm_), for each time point, by the ROI total fluorescence (background- and pre-photoactivation-corrected of the first frame after the photoactivation (FROI_t*(0)*_), according to the formula:
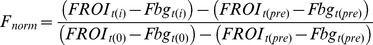



The data were then fitted (GraphPad Prism5 software) with a single exponential decay curve:
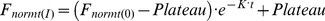
where Plateau is the F_norm_ value at infinite times and K is the rate constant (1/s). The time constant τ, expressed in (s), is the reciprocal of K. The half-life (t_1/2_) is ln(2)/K. For each experimental condition (normal medium, medium+DMSO or medium+Ika), the half-lives of each PAGFP-CFTR+adducin WT or PAGFP-CFTR+adducin G460W expressing cell were averaged and the different populations were compared using Student’s t-test. All data derived from three different independent experiments.

### Biotinylation Experiments

Forty-eight hours after the transient transfection of CFTR in the HU33 and NU12 cell lines, cells were biotinylated with 1 mg/ml EZ-Link sulfo-NHS-LC-biotin (Pierce). After blocking the biotinylation reaction in PBS/100 mM glycine buffer, cells were washed in PBS and lysed in Triton buffer (25 mM Tris-HCl, 150 mM NaCl pH 7.5, Triton 1%, BSA 0.2%). The biotinylated surface proteins were allowed to bind to streptavidin beads (Ultra link immobilized streptavidine, Pierce) for 24 hours at 4°C. After three washes in lysis buffer the proteins were released from the beads by incubation with 2× SDS loading buffer, separated by 6.5% SDS-PAGE and transferred onto PVDF membrane. The blots were probed for the expression of CFTR using the primary antibody anti-CFTR 24.1 (R&D Systems), and visualized by ECL.

### cAMP Assay

cAMP levels were determined using a cAMP-GLO assay kit (Promega) according to the manufacturers’ direction. Briefly, cells, seeded 5×10^3^/well in a 96 multiwell plate 24 hours before the assay, were washed with PBS and incubated in 20 µl of induction buffer containing 10 µM forskolin or DMSO (forskolin solvent, controls). Cells were then lysed in 20 µl of cAMP-GLO lysis buffer. Lysed cell and cAMP standards were transferred to a 96-well white wall plate, and, after a 20 min incubation in 40 µl of cAMP detection solution, 80 µl of Kinase GLO reagent were added. Luminescence was measured with a Tecan F200 Pro luminometer. cAMP concentration was calculated using the difference between Relative Luminescence Units (RLU) values of unstimulated and stimulated cells and the equation generated from a cAMP standard curve, following manufacturers’ instructions. The assays were performed on 4 independent series of samples, and for each series every tested condition was assayed on three distinct wells and luminescence data were normalized for protein concentration.

### Statistical Analysis

All the data are presented as mean ± SEM. Statistical analyses were performed using an unpaired Student’s t test or one-way or two way ANOVA (to analyse multiple data). Statistical assessments were done using the statistical package of Prism version 4 (GraphPad, San Diego, Calif.). The criterion for statistical significance was a P-value <0.05.

## Results

### Adducin Effects on CFTR Function: Patch-clamp Analysis in HEK Cells

**Figure 1:**
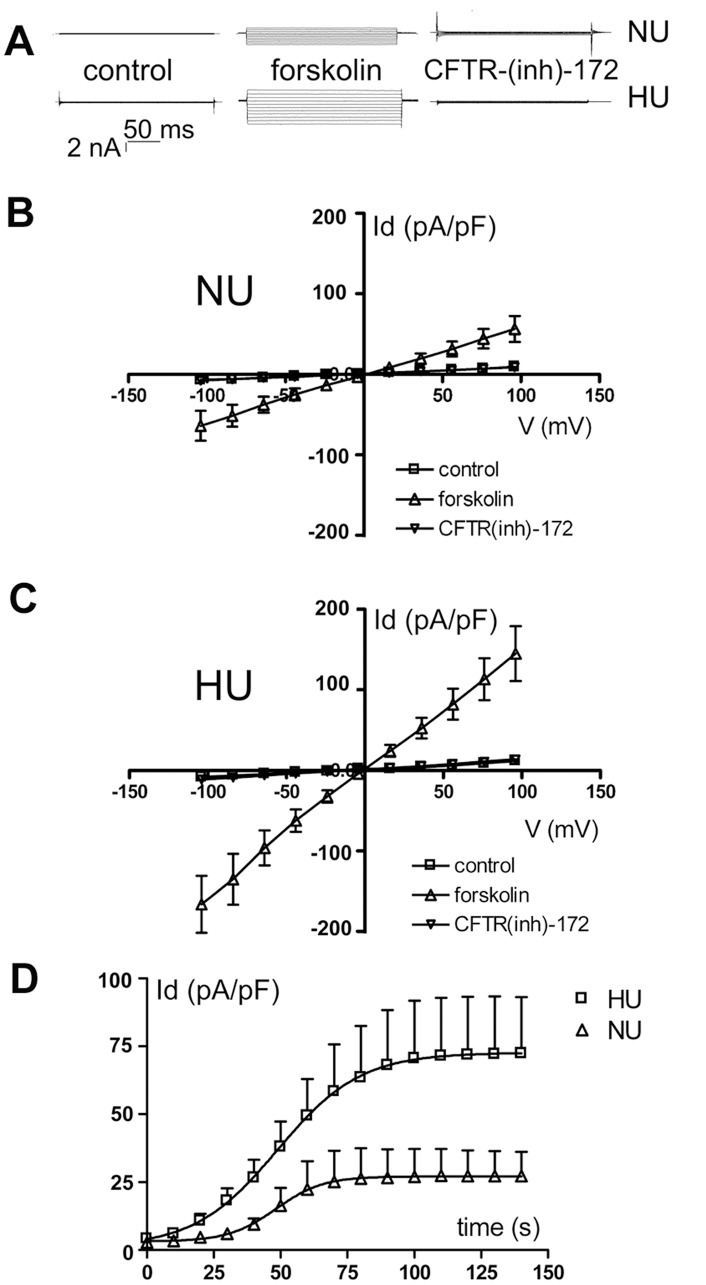
G460W adducin affects CFTR whole-cell current. Chloride currents in NU12 and HU33 cells overepressing the CFTR channel (patch-clamp, whole-cell configuration). A) Representative traces of the currents recorded in NU12 (*NU*) and HU33 (*HU*) cells in the presence of symmetrical solutions (*control*), after the exposure to 10 µmol/L forskolin, and to 2 µmol/L CFTR-(inh)-172+10 µmol/L forskolin. B) Current-density (pA/pF) to voltage (Id-V) relation recorded in NU12 cells (*NU*) transfected with the pIRES2-EGFP-CFTR vector. Whole-cell currents were recorded in the presence of symmetrical chloride solutions (n = 12), 140 s after forskolin exposure (n = 12), and after CFTR-(inh)-172 inhibition (n = 12). C) Id-V relation recorded in HU33 cells (*HU*) transfected with the pIRES2-EGFP-CFTR vector (n = 14). The current density was significantly higher after forskolin exposure in HU versus NU cells (P<0.01). D) Activation kinetics of the CFTR-mediated chloride current in NU12 and HU33 cells (*NU* and *HU*) transfected with the pIRES2-EGFP-CFTR vector. Activation currents values were interpolated by sigmoid curves, that were significantly different in HU33 versus NU12 cells (P<0.01).

To study whether the expression of adducin has functional consequences on CFTR activity, we performed whole-cell patch-clamp experiments (Figure 1) on NU12 (HEK cells stably transfected with the human wild-type adducin) and HU33 cells (expressing the G460W mutation), transiently transfected with a human CFTR expressing vector (pIRES2-EGFP-hCFTR) or with the control pIRES2-EGFP vector (data not shown). In the presence of symmetrical chloride solutions ([Cl^−^] = 153 mmol/L) in the pipette and bath solution, current density Id (pA/pF, i.e. the current intensity normalized for the cell capacitance, an index of cell size) was not significantly different between NU12 and HU33 cells. After exposing cells to 10 µmol/L forskolin, that activates CFTR currents by raising the levels of cyclic AMP [35], a Cl^−^ current was observed in the CFTR overexpressing cells, but not in the cells transfected with the control vector (data not shown). At maximal activation, the Id of the forskolin activated current was significantly higher in HU33 than in NU12 cells (P<0.01). In both cell types, the current - voltage relationship was linear and the reversal potential (E_rev_) was not significantly different from 0 mV (NU12: 0.84±0.4 mV, n = 12; HU33: 1.6±0.3 mV, n = 14), as expected for a chloride current in our experimental conditions (absence of Cl^−^ chemical gradient). The current was inhibited by 2 µmol/L CFTR-(inh)-172, a specific inhibitor of CFTR channel activity [36], to a value not significantly different from the value recorded before forskolin exposure (Figure 1B–C).

The current activation curve (Figure 1D) was well fitted (least squared method) by a sigmoid (R^2^ = 0.9992 and 0.9998 in NU12 and HU33 cells respectively). The slope of the regression was significantly higher (P<0.01) in HU33 (0.049±0.0019 pA×pF^−1^×s^−1^, n = 14), compared with NU12 cells (0.028±0.005 pA×pF^−1^×s^−1^, n = 12), whereas the t_50_ (time to reach the half-maximal activation) was not significantly different between the two curves (48.78±0.34 s for NU12 and 48.29±0.40 s for HU33 cells).

**Figure 2:**
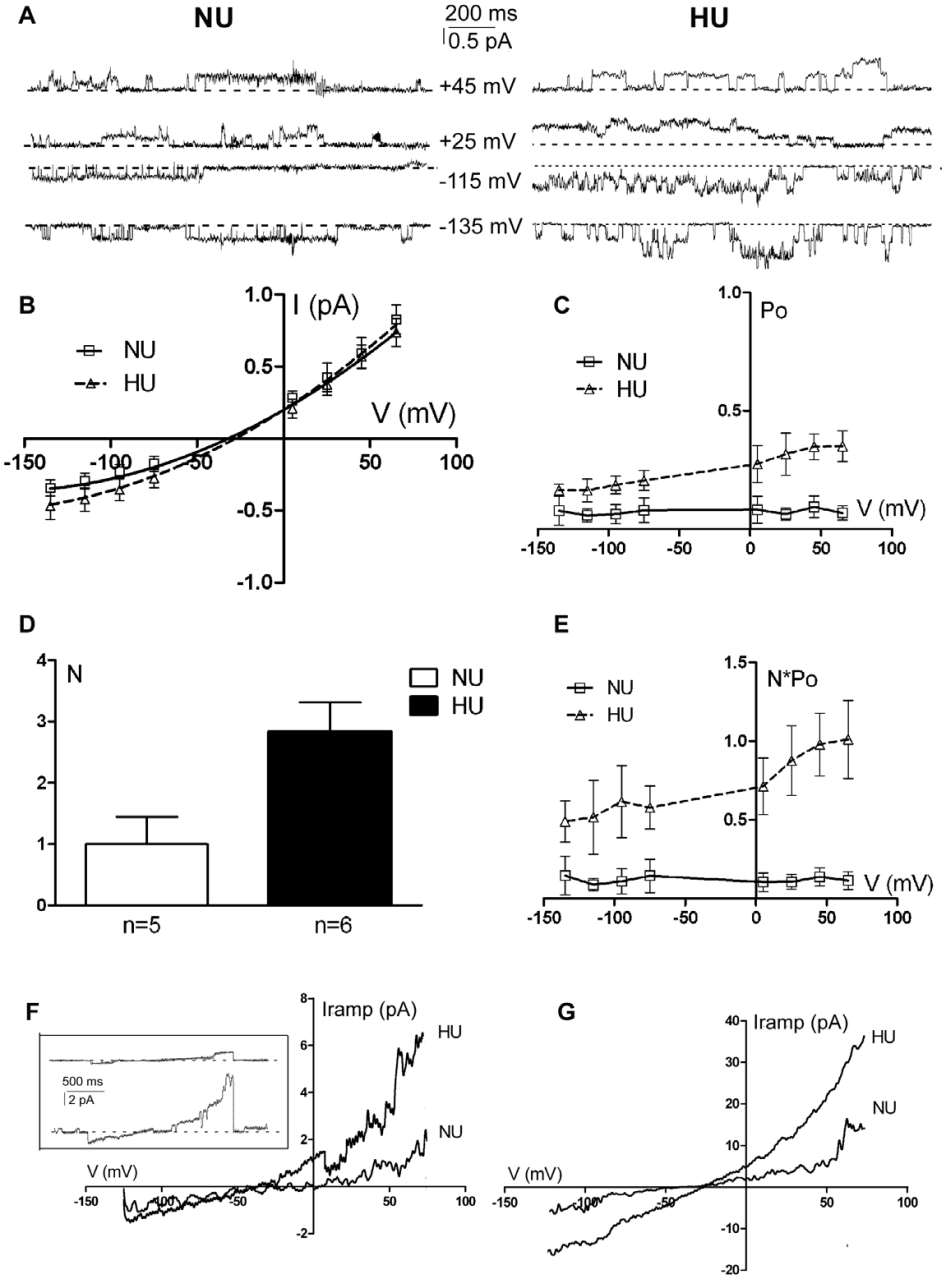
G460W adducin affects CFTR single channel activity. Chloride currents in NU12 and HU33 cells overexpressing the CFTR channel (patch-clamp, cell-attached configuration). A) Representative single-channel traces at positive or negative potentials as indicated, recorded in NU12 (*NU*, left panel) or HU33 cells (*HU*, right panel). The dotted line corresponds to the closed level. B) Single channel current-voltage (I–V) relation for CFTR in NU12 and HU33 cells (n = 5 and 6). C) Open probability (Po) versus potential plots for CFTR channels recorded in NU12 (n = 5) and HU33 cells (n = 6). D) Channel density in NU12 (n = 5) and HU33 (n = 6) cells (*N* = channel density, *n* = total number of seals). P<0.05. E) Channel activity, measured as N multiplied by channel open probability (Po), versus membrane potential for NU12 (n = 5) and HU33 (n = 6) cells. P<0.05. F) Mean current recorded during voltage protocol consisting of ramps from −135 mV to +65 mV, applied to cell-attached patches at a rate of 0.5 mV/ms. The I/V represents the mean current of 10 ramp protocols from 5 and 6 patch of NU12 and HU33 cells respectively. In the box representative single ramp records are reported for NU12 (upper ramp) and HU33 (lower ramp). G) Mean current recorded in the same condition as in F, after 3 minutes exposure to 10 µmol/L forskolin.

We also investigated adducin effect on CFTR single channel properties by performing cell-attached experiments on HU33 and NU12 cells transiently transfected with a pIRES2-EGFP-hCFTR expressing vector (Figure 2). In these experiments, differently from whole-cell experiments, the imposed potential differences were expressed as overall potential differences, determined by the algebraic sum of the junction potential (−5 mV), the holding potential (from −100 mV to +100 mV), and the cell membrane potential, about −30 mV (measured with current clamp experiments). A channel with a conductance of 10.5±0.9 pS (n = 6) and 10.8±0.6 pS (n = 5) was recorded in HU33 and NU12 cells, respectively (Figure 2B). This channel was never observed in non-transfected cells (n = 10) and had a voltage independent Po. The E_rev_ (the reversal potential, corrected for the junction potential and the membrane cell potential) was not statistically different between the two cell types (−31.4±8.1 mV (n = 6) and −31.0±5.2 mV (n = 5) in HU33 and NU12 cells respectively) and was compatible with that expected for a chloride permeable channel (Figure 2B–C). The channel I/V relationship showed a pronounced rectification, differently from what observed in whole-cell experiments, and this is presumably due to the asymmetrical chloride concentration in cell-attached condition ([Cl^−^]_in_ lower then [Cl^−^]_out_ = 151 mM). Channel density (number of active channels per patch, 2.8±0.5 channels/patch, n = 6 and 1.0±0.4 channels/patch, n = 5 in HU33 and NU12 cells respectively) and activity were significantly higher in HU33 than NU12 cells (Figure 2D–E). Accordingly, during voltage-ramp protocols, in which the voltage of the patch was linearly changed from −135 mV to +65 mV and allowed to directly generate current-voltage relations, the mean current (I_ramp_) recorded from patches containing one or more active channels was significantly lower (P<0.05) in NU12 than HU33 cells (Figure 2F). After 140 s of 10 µmol/L forskolin exposure, the I_ramp_ increased in both NU12 and HU33 cells but it was still significantly higher (P<0.05) in HU33 than NU12 cells (Figure 2G).

Since it has been reported that actin binding protein can directly or indirectly modulate adenylate cyclase activity [37], cAMP levels were assayed using a cAMP-GLO assay kit (Promega) in HU33 and NU12 cells both before and after the addition of forskolin 10 µM to the cell culture media. cAMP levels were not significantly higher in HU33 compared to NU12 both in the presence (217451.5±77983.31 RLU (n = 4) and 113667±25298.86 RLU (n = 4) in NU12 and HU33 cells respectively, P>0.05) or in the absence (370905.9±68154.9 RLU (n = 4) and 205104.7±25875.07 RLU (n = 4) in NU12 and HU33 cells respectively, P>0.05) of forskolin. Also cAMP concentration measured as difference between unstimulated and stimulated cells was not significantly different between the two cell types (0.024026±0.011712 nM (n = 4) and 0.014316±0.002341 nM (n = 4) in NU12 and HU33 cells respectively, P>0.05).

### CFTR Expression and Activity in MNS and MHS Cells

The data obtained with the transfected HEK cell line indicate that the G460W mutation of adducin induces an upregulation of CFTR activity, that is likely to be the consequence of an increased number of active channels. To verify whether this effect was detectable also in kidney cells isolated from an animal model, we performed patch-clamp experiments on distal convoluted tubule (DCT) primary cells derived from DCTs isolated from Milan hypertensive rats (MHS), bearing a F316Y hypertensive α-adducin mutation, and from control normotensive rats (MNS) [38].

**Figure 3:**
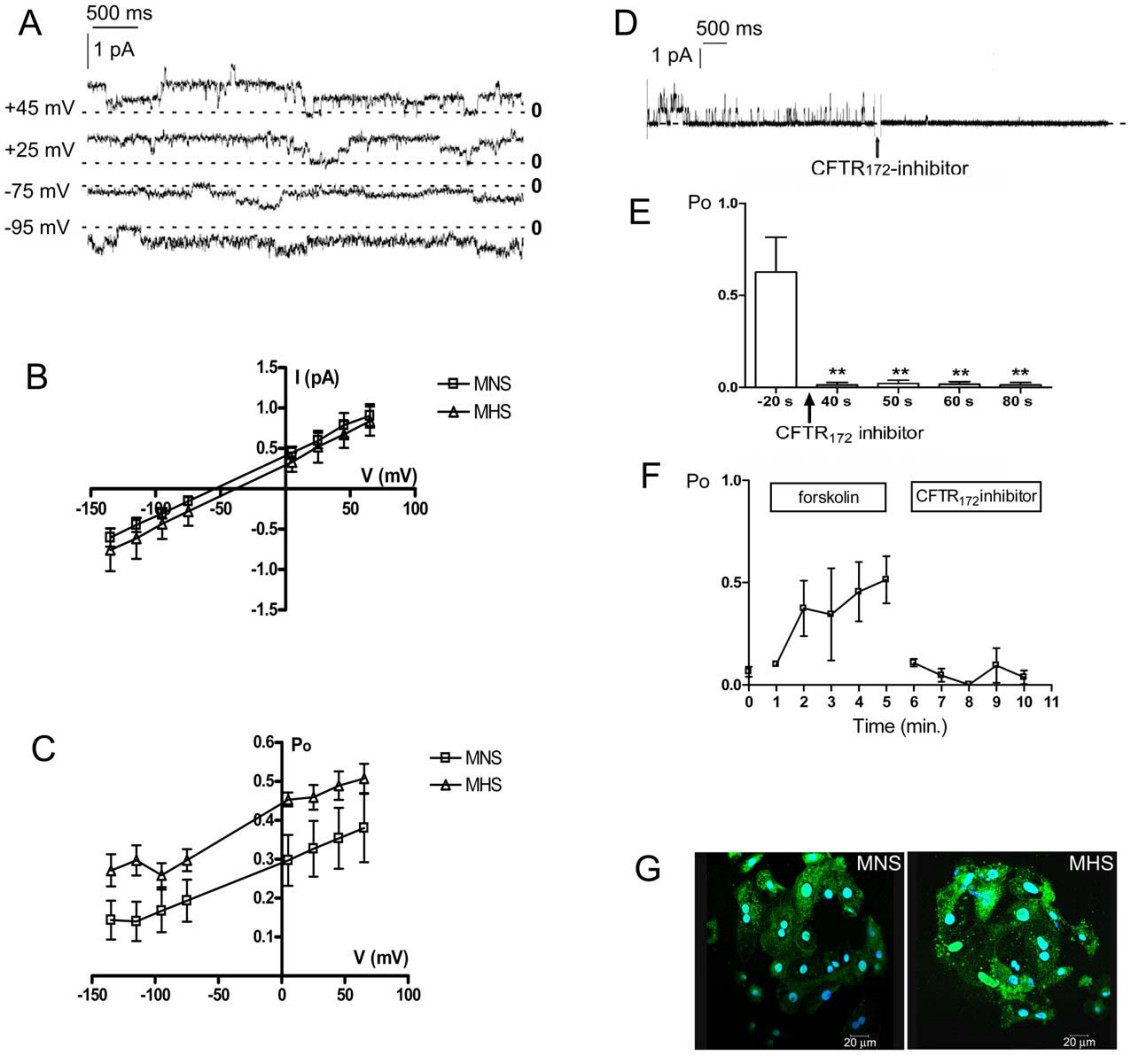
CFTR channel in MNS and MHS DCT cells. A) Representative traces of the rat 8 pS chloride channel recorded at positive or negative potentials in DCT cells isolated from 11 MHS and 16 MNS rats. 0 indicates the closed level. B) Mean current-voltage (*I-V*) curve of CFTR in MNS (n = 3) and MHS DCT cells (n = 8). C) Open probability (*Po*) curve of CFTR in MNS (n = 3) and MHS DCT cells (n = 8). D) Representative trace showing inhibition by CFTR-(inh)-172. E) Histograms representing the channel open probability (*Po*) before or after 2 µmol/L CFTR-(inh)-172 exposure (n = 4). **P<0.01. F) Channel open probability (*Po*) after 10 µmol/L forskolin and 2 µmol/L CFTR-(inh)-172 exposure (n = 4). G) Confocal images showing CFTR expression in MNS and MHS DCT cells. CFTR is located both in the plasma membrane and in intracellular vesicles. Primary antibody: anti-CFTR; secondary antibody: Cy2 anti-rabbit. Blue: nuclei DAPI staining. Scale bar is 20 µm.

Cell-attached patch-clamp experiments were performed on cultured MHS and MNS primary DCT cells (Figure 3). In both cell types we observed a Cl^−^ channel (Figure 3B–C) characterized by a conductance of 7.45±0.88 pS (n = 3) in MNS and 8.4±0.6 pS (n = 8) in MHS cells. The current reversal potential (E_rev_) was −54.0±3.7 mV in MNS (n = 3) and −42.4±4.0 mV (n = 8) in MHS and the channel open probability (Po) was voltage-independent.

Further experiments were conducted to characterize this channel (Figure 3D–E). After exposure to 2 µmol/L thiazolidinone CFTR-(inh)-172 (Figure 3E) the Po was significantly reduced (P<0.01). In another set of experiments performed on selected patches in which the channel activity was constitutively low (Figure 3F), the Po increased from 0.03±0.01 (n = 4) to 0.49±0.12 (n = 4) after 5 min. exposure to 10 µmol/L forskolin. The forskolin-activated channel was inhibited by 2 µmol/L CFTR-(inh)-172.

This pharmacological profile, along with the channel biophysical properties, matched those of CFTR, whose expression was confirmed in the MNS and MHS DCT cells by immunocytochemical experiments (Figure 3G).

**Figure 4:**
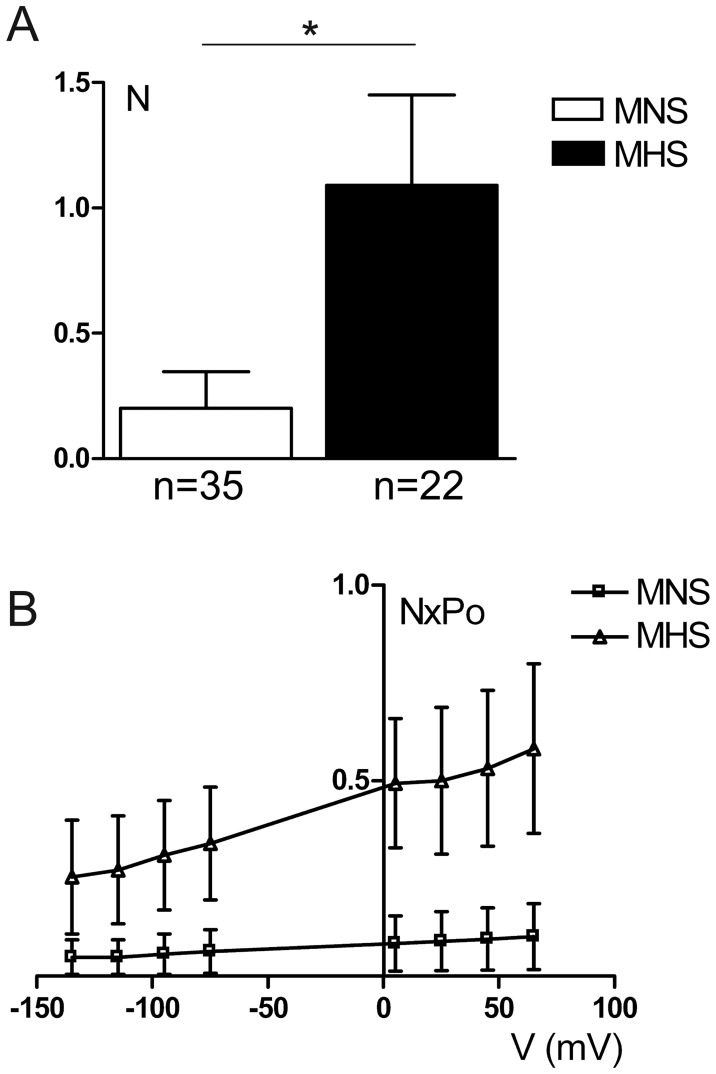
CFTR density and activity. A) Channel density in MNS (n = 35) and MHS (n = 22) DCT cells (*N* = channel density, *n* = total number of seals). *P<0.05. B) Channel activity (*NxPo,* measured as N multiplied by channel Po) versus membrane potential (*V*) for MNS (n = 35) and MHS (n = 22) channels. *P<0.05 when comparing the two data sets.

Similarly to what we found in HU33 and NU12 cells, even if single channel conductance, E_rev_ and Po did not change significantly between MHS and MNS cells, the channel density (Figure 4A) and activity (calculated as N^.^Po, Figure 4B) were significantly (P<0.05) increased in MHS rats.

### Adducin Effects on CFTR Surface Expression

**Figure 5:**
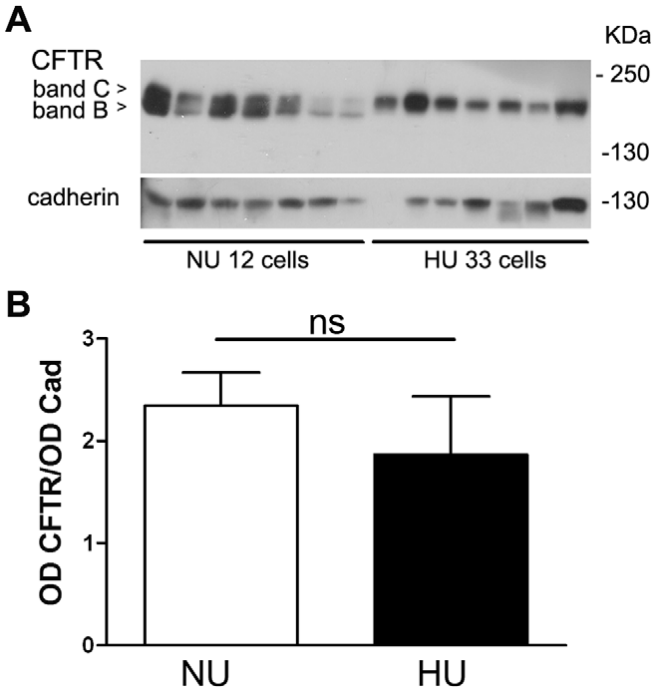
Expression of total CFTR protein (fully and core glycosylated) in NU12 and HU33 cells. A) Western blot analysis of CFTR expression in NU12 and HU33 cells transiently transfected with the pcDNA3-CFTR vector; arrows indicate the core-glycosylated (band B) and fully glycosylated (band C) forms of CFTR. The blot was probed with anti-CFTR antibody (top panel) and anti-cadherin antibody (lower panel). B) Densitometric analysis of the ratio of total CFTR (band C+band B)/cadherin band intensities (*OD CFTR/OD Cad*: n = 7 for NU12, n = 6 for HU33); *ns* = non significative. Only samples showing both CFTR and cadherin clear signals were considered for the quantitative analysis.

In both models of kidney cells we found that the expression of mutated adducin results in an upregulation of CFTR activity. To assess adducin effect on CFTR expression we performed western blot experiments on HU33 and NU12 cell lysates transiently transfected with CFTR (Figure 5). Cadherin was probed on the same blot to serve as loading control. CFTR antibody recognized the fully- (band C, 160 kDa) and core-glycosylated (band B, 140 kDa) proteins [39] in NU12 cells, while only the band C was clearly detectable in HU33 cells (Figure 5A). CFTR band pattern in NU12 cells was similar to that of HEK293 cells non transfected with adducin (Figure S2, panel C), suggesting that the stable over-expression of WT adducin does not *per se* affect CFTR maturation.

Densitometric analysis of the bands revealed that total CFTR (band B plus band C) was not significantly different in NU12 and HU33 cells (Figure 5B).

**Figure 6:**
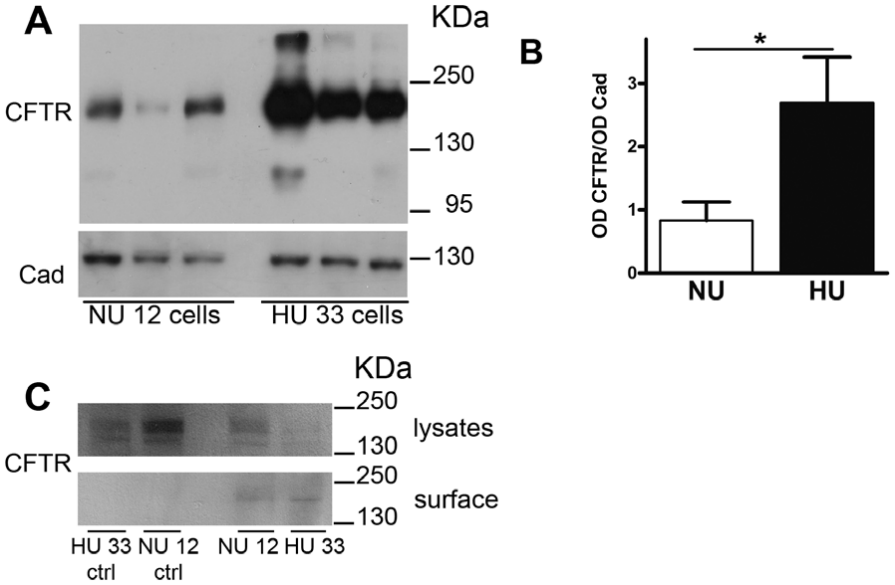
Expression of CFTR in the plasmamembrane fraction in NU12 and HU33 cells. A) Western blot analysis of CFTR expression in NU12 and HU33 cells transiently transfected with the pcDNA3-CFTR vector; only one band at a molecular weight compatible with that of band C (∼160 kDa) is visible in the plasmamembrane fraction. The blot was probed with anti-CFTR antibody (top panel) and anti-cadherin antibody (lower panel). B) Densitometric analysis of the ratio of plasmamembrane CFTR/cadherin band intensities (*OD CFTR/OD Cad*, n = 6). *P<0.05. C) Western blot analysis of biotinylated CFTR in NU12 and HU33 cells transiently transfected with the pcDNA3-CFTR vector. CFTR signal (anti-CFTR antibody) in cell lysates (*lysates*, upper panel) and in the cell surface fraction (*surface*, lower panel) are reported. Only one band compatible with band C is detectable in the cell surface fraction. No biotinylation condition served as control (*NU12 ctrl*, *HU33 ctrl*).

To further investigate whether the plasma membrane expression level of CFTR changed in the presence of the G460W adducin, we performed Western blot experiments on the sole plasmamembrane protein fraction of NU12 and HU33 cells (Figure 6). The purity of membrane preparations was checked by monitoring cadherin and calreticulin enrichment, as reported in Figure S2. Only one band, with a molecular weight compatible with that of band C, was revealed in both cell types (Figure 6A), but the protein abundance was significantly increased in HU33 cells (CFTR/cadherin  = 0.83±0.30, n = 6 and 6; 2.69±0.72, n = 6 in NU12 and HU33 respectively, Figure 6B). This effect was not depending on a difference in the transfection efficiency of HU33 and NU12 cells, as shown in Figure S2.

The increased quantity of CFTR in the plasmamembrane fraction was confirmed also by biotinylation experiments (Figure 6C).

### CFTR and Adducin Interaction

CFTR protein is known to interact with several cytoskeletal proteins that modulate its localization, expression, and function. To investigate the interaction between CFTR and wild type or mutated (G460W) α-adducin, we performed both FRET and immunoprecipitation experiments. The FRET efficiency was evaluated with the acceptor photobleaching method (Figure S3). Control experiments were executed on HEK293 T cells co-expressing ECFP and YFP-CFTR, a chimeric protein with EYFP fused to the N-terminal of CFTR, where it doesn’t interfere with CFTR localization and glycosylation pattern [40]. These experiments allowed to estimate the aspecific FRET efficiency (FRETeff %, in this case −0.43±0.47, n = 27, Figure S3C). In cells co-expressing YFP-CFTR and CFP-WT adducin (a chimeric protein with ECFP fused to the N-terminal of adducin), a low FRET signal, yet significantly higher than in control (P<0.01), was recorded (FRETeff%: 3.65±0.71, n = 40). The FRETeff % was not significantly different from that observed when the CFP-G460W adducin protein was expressed (FRETeff%: 3.46±0.45, n = 35). Since CFTR channel properties and expression are modulated by forskolin, the same experiments were also performed after cell exposure to the drug (Figure S3D), but no significant effect was observed on FRET efficiency.

The measured FRETeff% was small, even if significantly different from our control condition, therefore we further investigated the possible interaction between adducin and CFTR by immunoprecipitation experiments. These assays were performed by co-expressing in HEK293 T cells both CFTR and tagged-adducin (Figure S4), both with a C-terminal tag (adducin-FLAG, immunoprecipitated with an anti-FLAG antibody, Figure S4A) and with an N-terminal tag (HA-adducin, immunoprecipitated with an anti-HA antibody, Figure S4B). Anyway, in both conditions, no clear specific CFTR co-immunoprecipitation was evidenced.

### Adducin Influence on CFTR Trafficking

**Figure 7:**
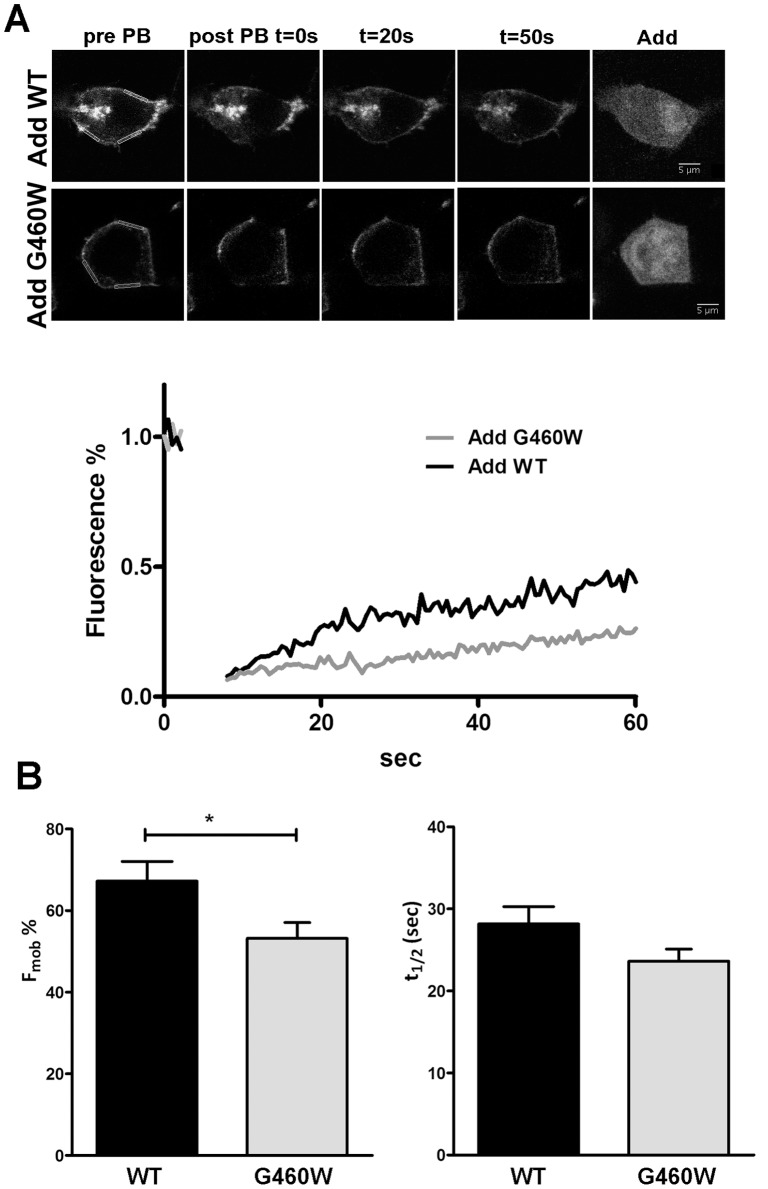
G460W adducin influences CFTR trafficking: FRAP experiments. A) Cell images from a FRAP experiment: YFP-CFTR signal in a HEK293 T cell over-expressing CFP-WT adducin (*Add-WT*, upper panels) or CFP-G460W adducin (*Add-G460W*, lower panels) before YFP photobleaching (*pre PB*) and at different time-points after the photobleaching (*post PB t = 0 s, 20 s, 50 s*); on the right the corresponding CFP-adducin signal (*Add*) is reported for each condition. In the pre-photobleaching images three membrane ROIs are shown. Scale bar is 5 µm. The graph shows the kinetics of CFTR-YFP fluorescence recovery after photobleaching in presence of the CFP-adducin WT or G460W mutated variant. B) Mean CFTR mobile fraction percentage (F_mob_%) and mean half-life constants (t_1/2_) of HEK cells over-expressing YFP-CFTR and CFP-WT adducin (n = 38) or CFP-G460W adducin (n = 42). *P<0.05.

CFTR function is correlated to its trafficking regulation. To investigate a possible interference of mutated adducin on CFTR trafficking and diffusional mobility in the plasma membrane, we performed FRAP experiments. HEK293 T cells were transiently transfected with YFP-CFTR and CFP-adducin (Figure 7) and YFP fluorescence recovery, after YFP-CFTR photobleaching, was followed in selected regions of interest (ROIs) comprising membrane regions (Figure 7A). Quantitative image analysis was used to determine the kinetics of fluorescence recovery after photobleaching (Figure 7A–B). The percentage fluorescence recoveries, generally interpreted as the fraction of mobile GFP-labeled molecules (F_mob_%), was 67.20±4.84 (n = 38 experiments on cells from 3 transfection batches) for YFP-CFTR in the presence of WT adducin, a value comparable to those already reported for overexpressed-CFTR [41]. The mobility is likely to reflect both CFTR recycling and membrane internal movements, since the recovery in the lateral portion of the selected ROIs was faster than that in the central part of the ROI (central t_1/2_ 28.88±3.28 s; lateral t_1/2_ 18.41±1.55 s, n = 26 experiments on cells from 3 transfection batches, P<0.01). The co-expression with YFP-CFTR of the G460W variant significantly reduced the F_mob_% (53,18±3,88, n = 42 experiments on cells from 3 transfection batches, P<0.05). The average values half-time constant (t_1/2_) for WT and G460W adducin were not significantly different (WT 28.17±2.11 s, n = 38 experiments on cells from 3 transfection batches; G460W 23.63±1.47 s, n = 42 experiments on cells from 3 transfection batches).

**Figure 8:**
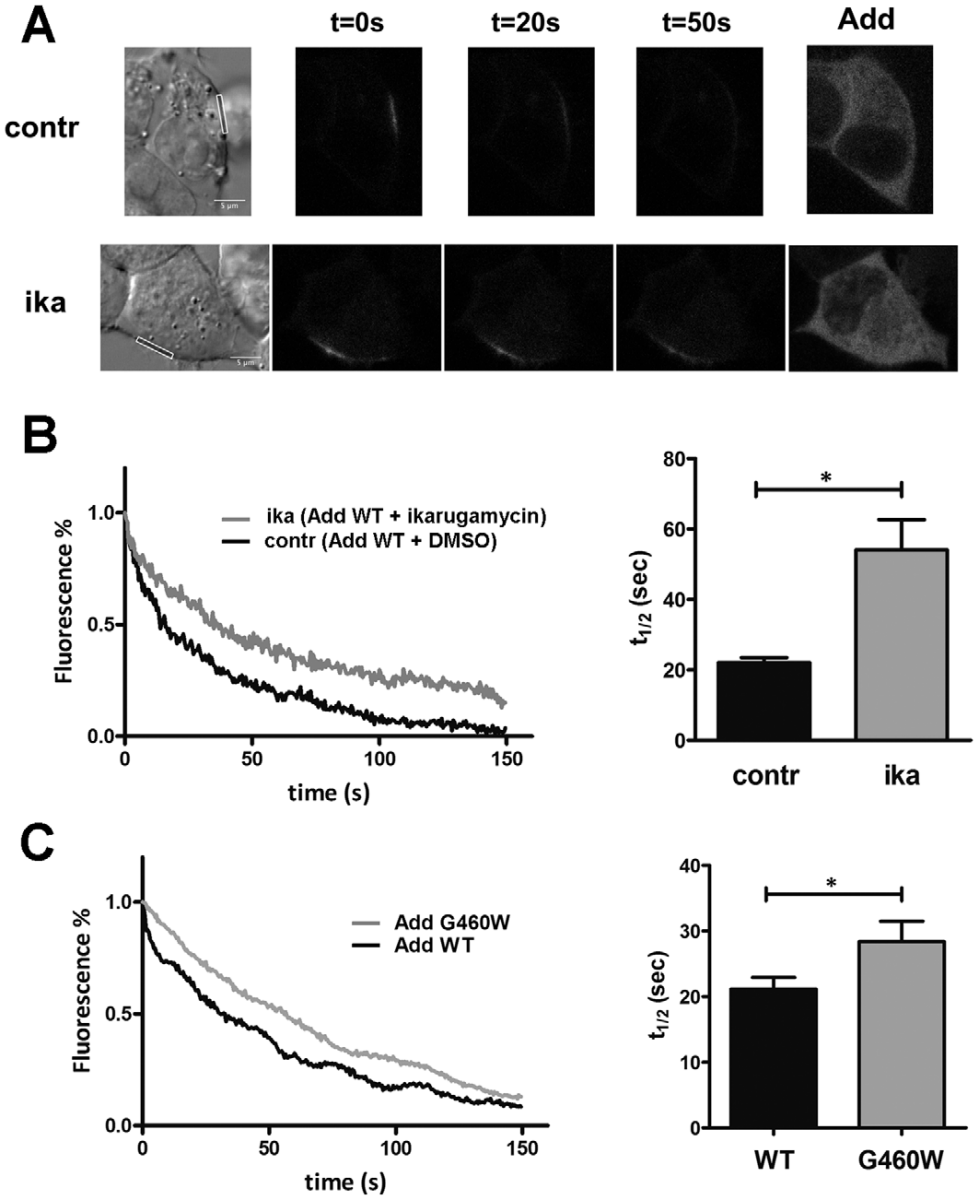
G460W Adducin influences CFTR trafficking: photoactivation experiments. A) Cell images from a photoactivation experiment: PAGFP-CFTR signal in a HEK293 T cell over-expressing CFP-WT adducin in control conditions (c*ontr*, upper panels) or in presence of ikarugamycin (*ika*, lower panels), an inhibitor of endocytotic recycling. Different time-points after photoactivation are shown (*t = 0 s, 20 s, 50 s*), together with the bright field images and the CFP-adducin signals (*Add*). In the bright field images the membrane ROIs are evidenced. Scale bar is 5 µm. B) Representative traces showing the kinetics of fluorescence decay after PAGFP-CFTR photoactivation in HEK cells over-expressing CFP-WT adducin exposed to ikarugamycin (*ika*, n = 19) or in control conditions (*contr*, n = 20). Mean half-lives (*t_1/2_*) are shown. *P<0.05. C) Representative traces showing the kinetics of fluorescence decay after PAGFP-CFTR photoactivation in HEK cells over-expressing CFP-adducin WT (*WT*, n = 30) or G460W mutated (*G460W*, n = 29) variant. Mean half-life constants (*t_1/2_*) are shown. *P<0.05.

To further investigate CFTR mobility and especially the influence of adducin on its trafficking we also performed photoactivation experiments, by transiently co-expressing PAGFP-CFTR (CFTR fused to a N-t photoactivable EGFP protein) and CFP-adducin (WT or G460W) in HEK cells. Also in this case we focused on the plasma membrane region, by photoactivating PAGFP-CFTR in ROIs comprising membrane portions and monitoring the decrease in time of the fluorescence (Figure 8A). The half time constant (t_1/2_), calculated by fitting the resulting curves with an exponential decay function, was 21.13±1.80 s (n = 30 experiments on cells from 3 transfection batches) in the presence of WT-adducin and was significantly (P<0.05) increased when G460W adducin was expressed (t_1/2_ = 28.35±3.12, n = 29 experiments on cells from 3 transfection batches, Figure 8C), thus confirming that the mutation of adducin causes a reduced CFTR mobility in the membrane region. The effect of G460W adducin on PA-CFTR mobility was similar, even if less intense, to that obtained by treating WT adducin transfected cells with ikarugamycin (Figure 8B, t_1/2_ = 22.07±1.40, n = 20 experiments on cells from 3 transfection batches for control; t_1/2_ = 54.04±8.57, n = 19 experiments on cells from 3 transfection batches for IKA; P<0.05), that acts as a general inhibitor of clathrin-coated pit-mediated endocytosis and has been used in endocytosis study [42], [43].

## Discussion

Several studies proved that CFTR activity is influenced by actin cytoskeleton and its polymerization state [26]. In this study we demonstrate that in G460W α-adducin expressing cells and in isolated DCT cells of MHS rats the activity of the CFTR channel increases as well.

The expression of the hypertensive mutation of adducin leads to a functional modification of CFTR activity, as observed in the patch-clamp experiments performed on HU33/NU12 stably transfected HEK cells. The forskolin activated whole-cell CFTR current recorded at maximal activation was significantly (P<0.05) higher in HU33 cells, expressing the hypertensive variant of adducin, than in NU12 cells, expressing the WT variant. The increase in current density at maximal activation, along with the observation that the slope of the activation, but not the t_50_, significantly increased, suggests a higher CFTR density in the plasmamembrane of G460W adducin expressing cells. The single channel experiments evidenced that the mutation of adducin associates with an increased channel activity both before and after forskolin exposure and CFTR activation. Measurements of intracellular cAMP level indicate that this is not due to an upregulation of the cAMP pathway in HU33 cells. Consistently with this hypothesis, the experiments performed on MNS/MHS rats indicate that this effect is reproducible also in cultured primary DCT cells, isolated from the animal hypertensive models. DCT is involved in hypertension maintenance in the MHS rats, in which the expression and activity of both Na^+^-Cl^−^ cotransporter and ClC-K increase [11], and the Na^+^/K^+^ pump is upregulated [12]. The experiments evidenced that the mutation of adducin in MHS rat DCT cells correlates with an increased CFTR channel density and activity, suggesting that CFTR could be directly or indirectly involved in the general alteration of renal ion transport in essential hypertension. Nowadays, despite the relatively high expression of CFTR in the kidney, its role in this organ is still elusive [19] and it has been hypothesized that CFTR could not only secrete but also absorb Cl^−^ across the apical membrane of distal tubules, at least when Na^+^ absorption is stimulated and the apical membrane potential depolarized [22], therefore contributing to the increased transcellular NaCl reabsorption observed in hypertension. It is possible that in the kidney its regulatory role might be more relevant than its activity as a chloride channel. It has recently been proposed that its membrane localization could influence both cytoskeletal organization and compartmentalization of signaling molecules such as cAMP and PKA in the subcortical compartment [44]. Moreover, CFTR is also known as a modulator of the activity of others transport systems, such as the ROMK K^+^ channel and the ENaC Na^+^ channel [19], [45]. Several studies have demonstrated in vivo an inverse relation between the putative CFTR levels and the expression of ENaC [46]. At a first glance, these observations are difficult to reconcile with the hypothesis that an increase of CFTR in the membrane would result in an increase of the NaCl intake, but could rather suggest that the increased surface expression of CFTR could help in counteracting the pathologically increment of salt uptake in the hypertensive subjects. Yet, the interaction between CFTR and ENaC seems to be more complex and dependent on the cell type: the activation of CFTR inhibits ENaC in airways cells [46], [47] and colonic cells [48], whereas it is accompanied by the concomitant activation of ENaC in absorptive sweat gland cells [49], in CCD kidney cells [50] and in MDCK cells [51]. As pointed out by Kunzelmann [52], the inhibitory effect of CFTR in some preparations may be due to the rise it produces in intracellular Cl^−^ concentration rather than to a direct molecular interaction of CFTR with ENaC. Therefore, in epithelial cells, that are actively involved in Cl^−^ secretion and absorption, and where chloride is maintained above its electrochemical equilibrium value, the effect of activating CFTR (e.g., by cAMP, forskolin, or cholera toxin) would result in a fall in intracellular Cl^−^ concentration, and therefore in ENaC activation [52]. In agreement with this observation, Xie and Shafer [51] demonstrated that the treatment with cAMP stimulates Cl^−^ secretion via cystic fibrosis transmembrane conductance regulator as well as ENaC-mediated Na^+^ absorption in MDCK cells. This could be in agreement with a lack of a kidney phenotype in cystic fibrosis patients [53], [54], in which CFTR activity is compromised, but chloride intracellular concentration could be modulated by the plethora of chloride channels [55] and transporters [56] that are active in kidney cells.

An involvement of CFTR in the renal regulation of extracellular fluid volume (ECFV) and, hence, in transepithelial transport, is additionally suggested by the observation that ECFV regulatory hormones, such as vasopressin or thyroid hormone, increase CFTR expression in the kidney [57], [58]. Actually it has already been proposed that an enhancement of CFTR activity could be involved in hypertension [54], [59], [60] and in hypertension related pathologies, such as Liddle syndrome [19], [50].

As a whole, additional studies are needed to better clarify the link between CFTR and kidney pathophysiology.

In this work we tried to further investigate the molecular mechanisms by which adducin could regulate CFTR activity.

In HEK cells overexpressing adducin, the measured increased activity of CFTR is paralleled by an altered processing efficiency of CFTR in the G460W adducin expressing cells; Western blot experiments revealed an increase of the ratio between the fully glycosylated band C and the core-glycosylated band B expression in HU33 compared with NU12 cells. The fully glycosylated band C of CFTR represents its mature form, predominantly located in the plasmamembrane and in the recycling submembrane vesicles, while band B represents the immature form of CFTR typical of the endoplasmic reticulum [39], [61]. The increase in the amount of plasmamembrane band C does not seem to be a consequence of a change in the total amount of CFTR (band B+C, Figure 5) and could be either due to an increase in the glycosylation efficiency or to a reduction of endocytosis, leading to a reduced turnover and to a longer retention of mature CFTR into the membrane. Channel glycosylation influences CFTR turnover in the post-endoplasmic reticulum compartments, with the fully glycosylated channel being more stable [62], [63], whereas it does not influence channel activity [62]. Therefore the enhancement of channel glycosylation observed in HU33 cells could lead to the observed increase of channel surface expression, revealed both by the Western blot experiments performed on the plasma membrane protein fraction and by biotinylation experiments (Figure 6), resulting in an increased number of active channels. Anyway, while no reports link adducin to glycosylation pathways, it has already been demonstrated that the expression of the mutated adducin reduces the endocytosis of Na^+^/K^+^-ATPase [8], [14] and of AQP4 water channel [64], suggesting that a similar mechanism is more likely to be directly involved also in the observed enhancement of CFTR surface expression and channel density.

To further investigate this point, we focused on CFTR-α adducin interaction and CFTR trafficking.

FRET experiments demonstrated a faint but significative FRETeff that might imply a close vicinity (in the nm range) between CFTR and adducin. The FRETeff was not significantly changed in the presence of the mutated G460W adducin variant both in the basal state or after the channel activation with forskolin. Anyway, immunoprecipitation experiments failed to reveal a clear co-immunoprecipitation of CFTR with adducin, suggesting that, if any, the possible interaction between the two proteins is likely to be feeble and unstable as already reported for the interaction between CFTR and actin mediated by intermediate cytoplasmic proteins [41].

Direct binding is not the only way by which adducin could modulate CFTR channel activity and affect its surface expression. CFTR, as adducin, forms a submembrane complex with several proteins, comprising actin [5], [35] or AP-2 [14], [65]. Adducin mutations could act by altering CFTR traffic, both through the modulation of cytoskeleton dynamics [26] and/or through affecting of AP2-μ2 phosphorylation [65], as already reported for the Na^+^/K^+^ pump [8], [14]. To investigate this point we performed both FRAP and photoactivation experiments. In both cases we found a rather high mobility of CFTR in the ROIs comprising plasmamembrane portions (70–52% F_mob_% for FRAP), with a comparable t_1/2_. The values of F_mob_% are similar to those obtained in other studies [41], [66] and the relatively high mobility, especially of WT-CFTR, is likely to be a consequence of overexpression, as already proposed [66], [67]. Moreover, at least part of the measured CFTR mobility is depending on lateral membrane diffusion, as proven, in the case of the FRAP experiments, by the lower t_1/2_ measured for the lateral portions of the ROIs, compared to the central ones. Yet, both FRAP and photoactivation experiments indicate that CFTR is more and/or longer retained in the membrane area when mutant adducin is co-expressed, according to the hypothesis that the mutations of adducin alter CFTR membrane mobility and apparently lower its membrane turnover. The effect of G460W adducin on PA-CFTR t_1/2_ was similar, even if less intense, to that obtained by treating WT adducin transfected cells with ikarugamycin (Figure 8B), suggesting that clathrin-coated pit-mediated endocytosis could be negatively affected by the mutation of adducin, as already reported for other transporters. The experiments cannot anyway exclude that also an altered actin cytoskeleton architecture might be involved. Actually, explorative immunofluorescence experiments confirm, in accordance with other reports [9], that the cytoskeleton of HEK cells seems to be affected by the expression of mutated adducin (Figure S5), presenting thicker and more abundant actin fibers, suggesting an increased cytoskeletal stiffness. Subcortical actin cytoskeletal is believed to be important for the anchoring of membrane proteins, thus limiting random lateral diffusion in the membrane, helping compartmentalization and also influencing the activity of ion channels and transporters [9], [44], [68]. Since CFTR is known to be subjected to clustering [69] and retention in zones of transient confinement [66], the state of the actin network is likely to impact on its mobility. Therefore, the retention of CFTR in the membrane area might be the consequence both of a reduced endocytosis and of an increased actin cytoskeletal stiffness induced by the mutated forms of adducin, that could exert an inhibitory effect on membrane protein lateral mobility [70], [71] and traffic [8], [72].

In conclusion, despite several hypotheses concerning CFTR involvement in hypertension, our study is, to our knowledge, the first to demonstrate a connection between CFTR and adducin, showing that channel activity is increased in isolated DCT cells of hypertensive MHS rats and in HEK cells overexpressing an hypertensive variant of adducin. In renal cell models, adducin mutations are likely to influence CFTR activity by affecting its membrane turnover, leading to a retention of the channel in the plasmamembrane.

Since CFTR is known to modulate the activity of many others transport systems, the increased surface expression of the channel could have consequences on the whole network of transport in the kidney cells. Further studies and additional analyses, either genetic or functional, on hypertensive patients as well, will help to fully understand the importance of CFTR in the modification of renal NaCl absorption *in vivo.*

## Supporting Information

Figure S1 **Adducin expression in NU12 and HU33 cells.** A) Western blot analysis of HA-adducin expression in NU12 (*Nu_1_*, *Nu_2_*) and HU33 (*Hu_1_*, *Hu_2_*) cells, compared with non transfected HEK cells (*NT_1_*, *NT_2_*). Two independent preparations are shown for each condition. The blot was probed with anti-HA antibody. B) Confocal images showing HA-adducin expression in NU12 (*NU*) and HU33 (*HU*) cells, together with bright field images. Primary antibody anti-HA, secondary antibody Alexa 488 anti-mouse; in the control condition (*contr*) the primary antibody was omitted. Scale bar is 10 µm.
(TIF)

Figure S2 **Assessment of HU33/NU12 transfection efficiency and plasma membrane protein enrichment.** A) Confocal images showing CFTR expression in HU33 (upper panels) and NU12 (lower panels) cells, transfected with pcDNA3-CFTR plasmid (*HU CFTR*, *NU CFTR,* left), non transfected NU and HU cells (*HU NT*, *NU NT,* right) served as control. Primary antibody: anti-CFTR, secondary antibody: Alexa 568 anti-mouse; nuclei are stained in blue (DAPI). Scale bar is 10 µm. Histograms illustrate the percentage of transfected NU12 (*NU*) and HU33 (*HU*) cells; showing no significant differences in transfection efficiency (n = 11). B) Transfection efficiency was also assessed with the Renilla luciferase reporter assay. NU12 and HU33 cells were cotransfected with pcDNA3-CFTR and pRL-TK (renilla luciferase reporter gene under the control of thymidine kinase promoter) plasmids, thus allowing the evaluation of cells transfection efficiency. The two histograms show luciferase activity normalized for the number of cells (*AU luminometry/N cells*, left, n = 15) or the total protein amount (*AU luminometry/mg prot*, right, n = 20) in NU12 (*NU*) and HU33 (*HU*) cells; no significant differences between the two cell types are revealed. C) Western blot analysis of CFTR expression in NU12 (*NU12*), stably transfected with WT adducin and control HEK293 (*HEK293*) cells, non transfected with adducin. Both cell types were transiently transfected with the pcDNA3-CFTR vector and probed with an anti-CFTR antibody. Both the 140 kDa core-glycosylated (band B) and the 160 kDa fully glycosylated (band C) forms of CFTR are detectable in the two cell types. D) Western blot analysis to assess plasma membrane separation efficiency. Western blot shows cadherin (plasma membrane marker; upper blot) enrichment in the plasma membrane, compared to calreticulin (endoplasmatic reticulum marker; lower blot). The upper blot was probed with anti-cadherin antibody, the lower blot was probed with anti-calreticulin antibody. The same quantity of proteins (25 µg) was loaded for all fractions. *PM*: plasmamembranes, *Lys*: lysate, *Cyt*: cytoplasmic fraction; *LP*: lower phase, formed by the remaining total membrane proteins, after the extraction of the plasma membrane fraction.
(TIF)

Figure S3 **Interaction between adducin and CFTR: FRET experiments.** The acceptor photobleaching method was used to obtain the FRET efficiency. A) Cell images of the CFP (*CFP*, upper panel) or YFP (*YFP-CFTR*, lower panel) channel before (*pre-bleach*) and after (*post-bleach*) the YFP photobleaching and FRET efficiency images (*FRET*) for HEK cells overexpressing CFP and YFP-CFTR (control). Scale bar is 10 µm. B) Cell images of the CFP (*Add-CFP*, upper panel) or YFP (*YFP-CFTR,* lower panel) channel before and after the YFP photobleaching and FRET efficiency images (*FRET*) for HEK cells overexpressing CFP- adducin WT and YFP-CFTR. Scale bar is 10 µm. C) FRET efficiency (*FRETeff %*) measured in control cells (*CFP+Y-CFTR*, n = 27) and in cells overexpressing YFP-CFTR and the CFP-adducin WT (*Y-CFTR+C-Add WT*, n = 40) or the G460W mutated variant (*Y-CFTR+C-AddG460W*, n = 35). **P<0.01. D) FRET efficiency (*FRETeff %*) measured after 10 µmol/L forskolin exposure in control cells (*CFP+Y-CFTR*, n = 4) and in cells overexpressing YFP-CFTR and the CFP-adducin, WT (*Y-CFTR+C-Add WT*, n = 11) or G460W mutated variant (*Y-CFTR+C-AddG460W*, n = 9). *P<0.05.
(TIF)

Figure S4 **Interaction between adducin and CFTR: immunoprecipitation experiments.** A) Immunoprecipitation experiment on HEK cells cotransfected with pcDNA3-CFTR and pcDNA3.1-WT adducin-FLAG (C-terminal FLAG tag, *Add-FLAG*); pcDNA3-CFTR and pFLAG-CMV4-BAP (bovine alkaline peroxidase, *FLAG-BAP*) cotransfection served as control. CFTR signal (upper panel; anti-CFTR antibody) and FLAG signal (lower panel; anti-FLAG antibody) in cell lysates (*Lys*), flow through (*FT*) and five sequential 40 µl eluates (*E1–E5*) are shown. B) Immunoprecipitation experiments on HEK cells cotransfected with pcDNA3-CFTR and pcDNA3.1-HA-WT adducin (N-terminal HA-tag, *HA-Add*). pcDNA3-CFTR and empty pFLAG-CMV4 plasmid cotransfection served as control (*ctrl*). CFTR signal (upper panel; anti-CFTR antibody) and HA signal (lower panel; anti-HA antibody) in cell lysates (*Lys*) and eluates (*E*) from 2 and 24 hours incubation with anti-HA agarose affinity gel are shown. Subscripts A and B refer to two independent preparations.
(TIF)

Figure S5 **Actin and adducin staining in NU12 and HU33 cells.** Confocal images showing actin (*Act*, left) and adducin (*Add*, right) in HU33 (*HU*, upper panels) and NU12 (*NU*, lower panels) cells. Primary antibody: anti-HA; secondary antibody: Alexa 488 anti-mouse. Actin fibers were stained with Alexa 568 Phalloidin. Scale bar is 10 µm.
(TIF)

Text S1 **Supplementary methods.** Additional methodological information concerning the results presented in the supplementary figures, i.e. evaluation of transfection efficiency, fluorescence resonance energy transfer (FRET) experiments, immunoprecipitation, HA-adducin and actin staining of HU33/NU12 cells.
(DOC)
