# Supplementary figures and images for: Hypertension-Linked Mutation of α-Adducin Increases CFTR Surface Expression and Activity in HEK and Cultured Rat Distal Convoluted Tubule Cells
Source: PLoS One. 2012 Dec 21;7(12):e52014. doi: 10.1371/journal.pone.0052014 (PMC3528715; doi:10.1371/journal.pone.0052014)

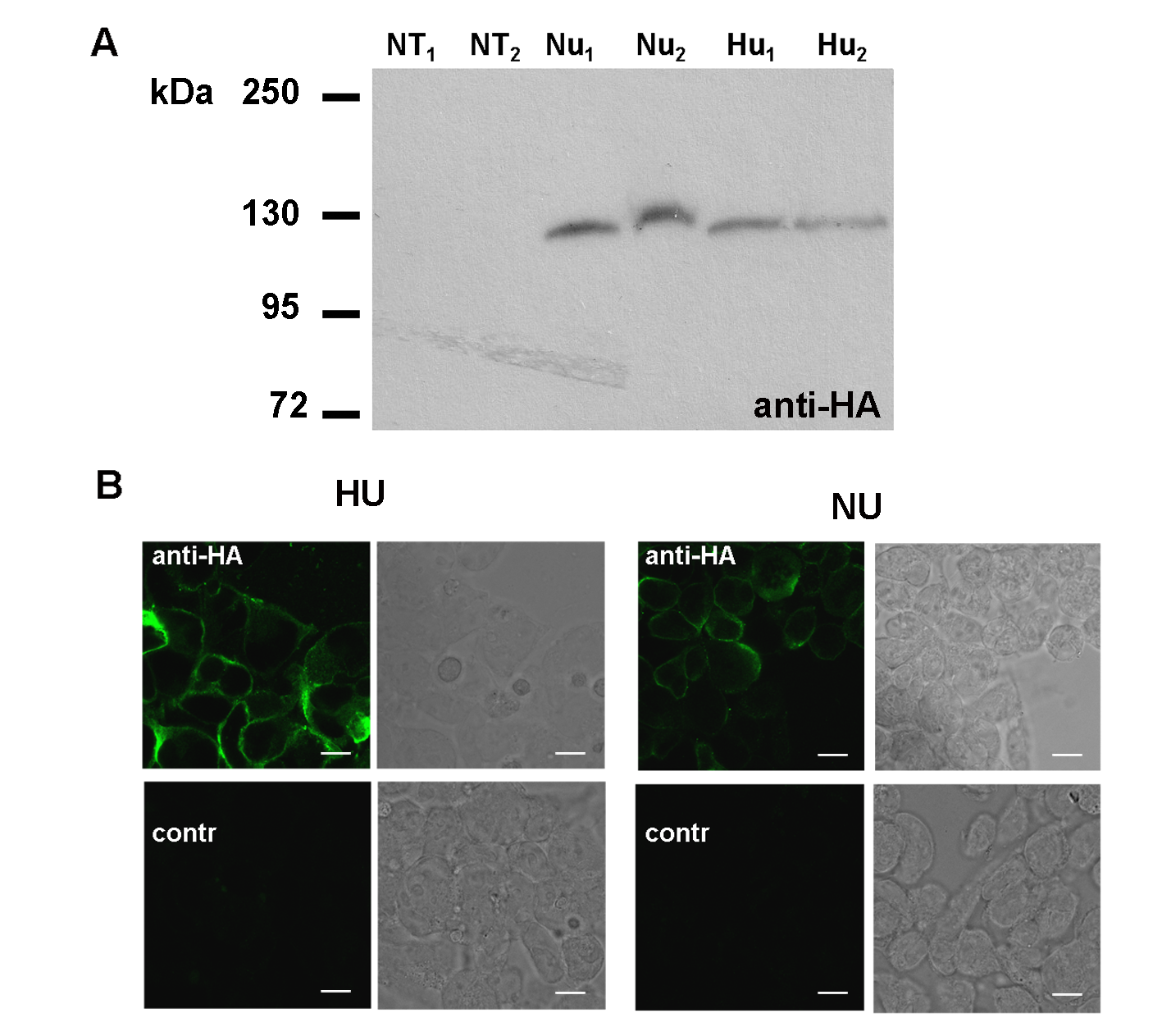

Supplement: Figure S1 — Adducin expression in NU12 and HU33 cells. A) Western blot analysis of HA-adducin expression in NU12 (Nu1, Nu2) and HU33 (Hu1, Hu2) cells, compared with non transfected HEK cells (NT1, NT2). Two independent preparations are shown for each condition. The blot was probed with anti-HA antibody. B) Confocal images showing HA-adducin expression in NU12 (NU) and HU33 (HU) cells, together with bright field images. Primary antibody anti-HA, secondary antibody Alexa 488 anti-mouse; in the control condition (contr) the primary antibody was omitted. Scale bar is 10 µm. (TIF) [file pone.0052014.s001.tif]

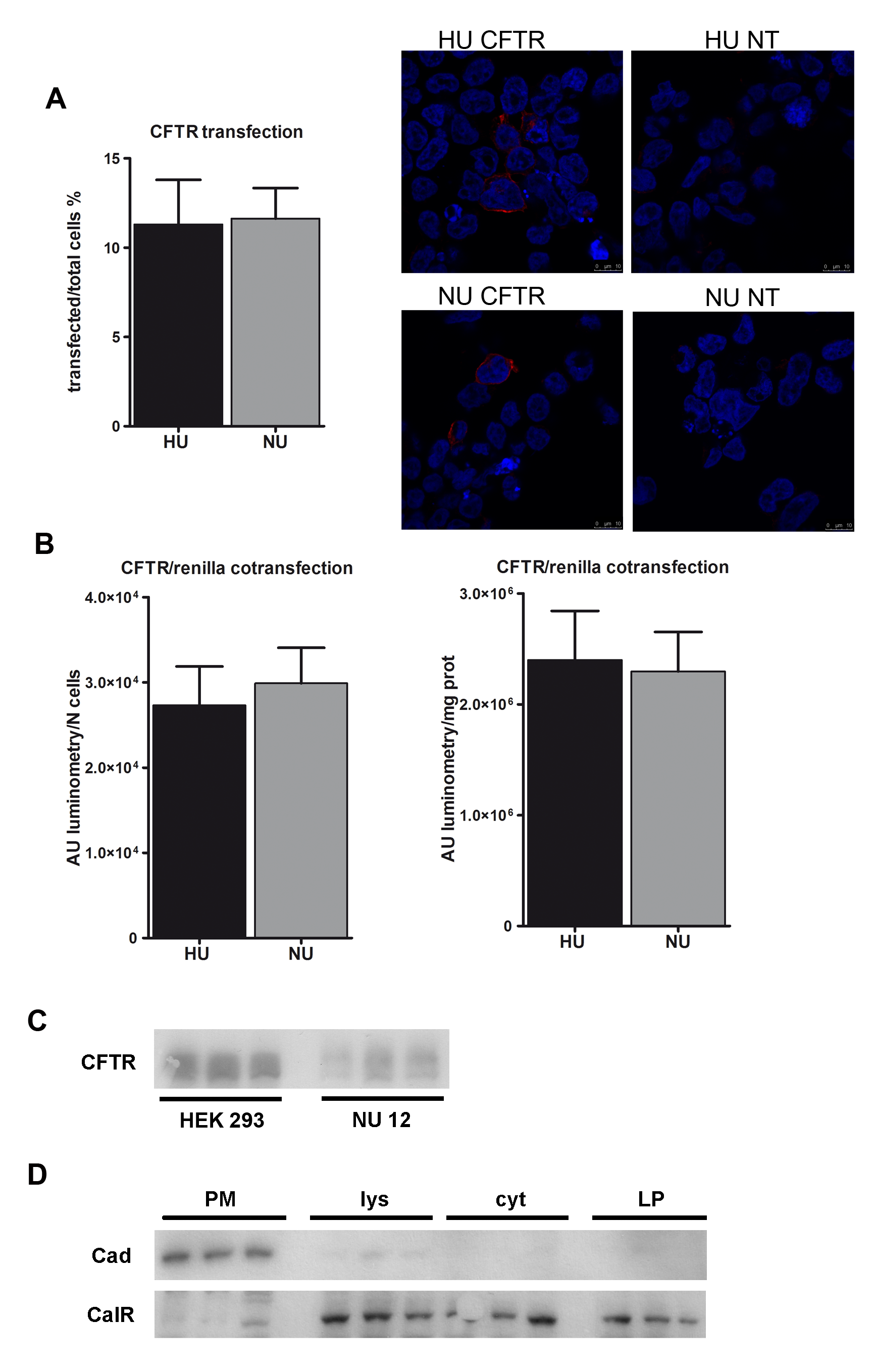

Supplement: Figure S2 — Assessment of HU33/NU12 transfection efficiency and plasma membrane protein enrichment. A) Confocal images showing CFTR expression in HU33 (upper panels) and NU12 (lower panels) cells, transfected with pcDNA3-CFTR plasmid (HU CFTR, NU CFTR, left), non transfected NU and HU cells (HU NT, NU NT, right) served as control. Primary antibody: anti-CFTR, secondary antibody: Alexa 568 anti-mouse; nuclei are stained in blue (DAPI). Scale bar is 10 µm. Histograms illustrate the percentage of transfected NU12 (NU) and HU33 (HU) cells; showing no significant differences in transfection efficiency (n = 11). B) Transfection efficiency was also assessed with the Renilla luciferase reporter assay. NU12 and HU33 cells were cotransfected with pcDNA3-CFTR and pRL-TK (renilla luciferase reporter gene under the control of thymidine kinase promoter) plasmids, thus allowing the evaluation of cells transfection efficiency. The two histograms show luciferase activity normalized for the number of cells (AU luminometry/N cells, left, n = 15) or the total protein amount (AU luminometry/mg prot, right, n = 20) in NU12 (NU) and HU33 (HU) cells; no significant differences between the two cell types are revealed. C) Western blot analysis of CFTR expression in NU12 (NU12), stably transfected with WT adducin and control HEK293 (HEK293) cells, non transfected with adducin. Both cell types were transiently transfected with the pcDNA3-CFTR vector and probed with an anti-CFTR antibody. Both the 140 kDa core-glycosylated (band B) and the 160 kDa fully glycosylated (band C) forms of CFTR are detectable in the two cell types. D) Western blot analysis to assess plasma membrane separation efficiency. Western blot shows cadherin (plasma membrane marker; upper blot) enrichment in the plasma membrane, compared to calreticulin (endoplasmatic reticulum marker; lower blot). The upper blot was probed with anti-cadherin antibody, the lower blot was probed with anti-calreticulin antibody. The same quantity of protein [file pone.0052014.s002.tif]

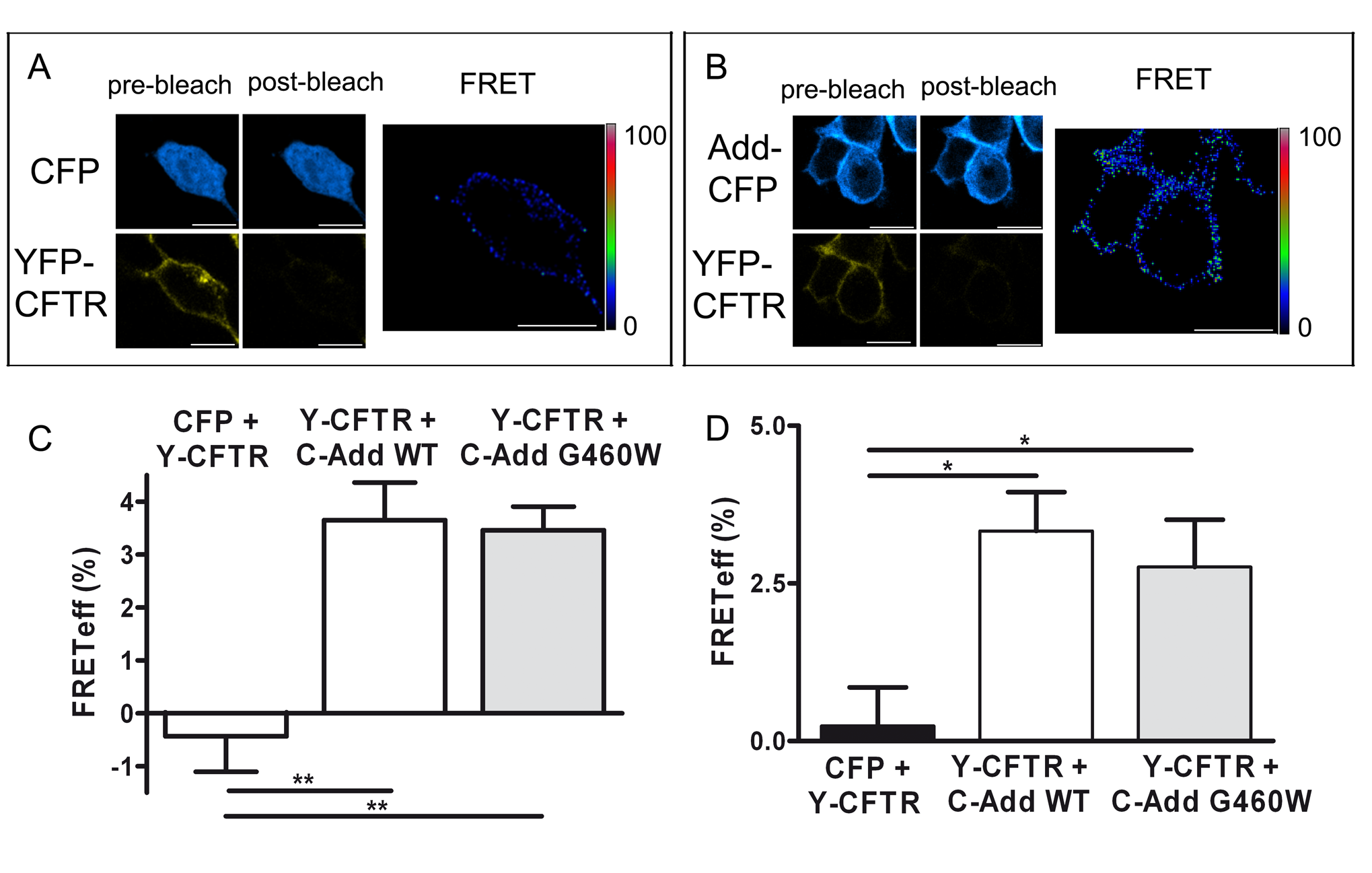

Supplement: Figure S3 — Interaction between adducin and CFTR: FRET experiments. The acceptor photobleaching method was used to obtain the FRET efficiency. A) Cell images of the CFP (CFP, upper panel) or YFP (YFP-CFTR, lower panel) channel before (pre-bleach) and after (post-bleach) the YFP photobleaching and FRET efficiency images (FRET) for HEK cells overexpressing CFP and YFP-CFTR (control). Scale bar is 10 µm. B) Cell images of the CFP (Add-CFP, upper panel) or YFP (YFP-CFTR, lower panel) channel before and after the YFP photobleaching and FRET efficiency images (FRET) for HEK cells overexpressing CFP- adducin WT and YFP-CFTR. Scale bar is 10 µm. C) FRET efficiency (FRETeff %) measured in control cells (CFP+Y-CFTR, n = 27) and in cells overexpressing YFP-CFTR and the CFP-adducin WT (Y-CFTR+C-Add WT, n = 40) or the G460W mutated variant (Y-CFTR+C-AddG460W, n = 35). **P<0.01. D) FRET efficiency (FRETeff %) measured after 10 µmol/L forskolin exposure in control cells (CFP+Y-CFTR, n = 4) and in cells overexpressing YFP-CFTR and the CFP-adducin, WT (Y-CFTR+C-Add WT, n = 11) or G460W mutated variant (Y-CFTR+C-AddG460W, n = 9). *P<0.05. (TIF) [file pone.0052014.s003.tif]

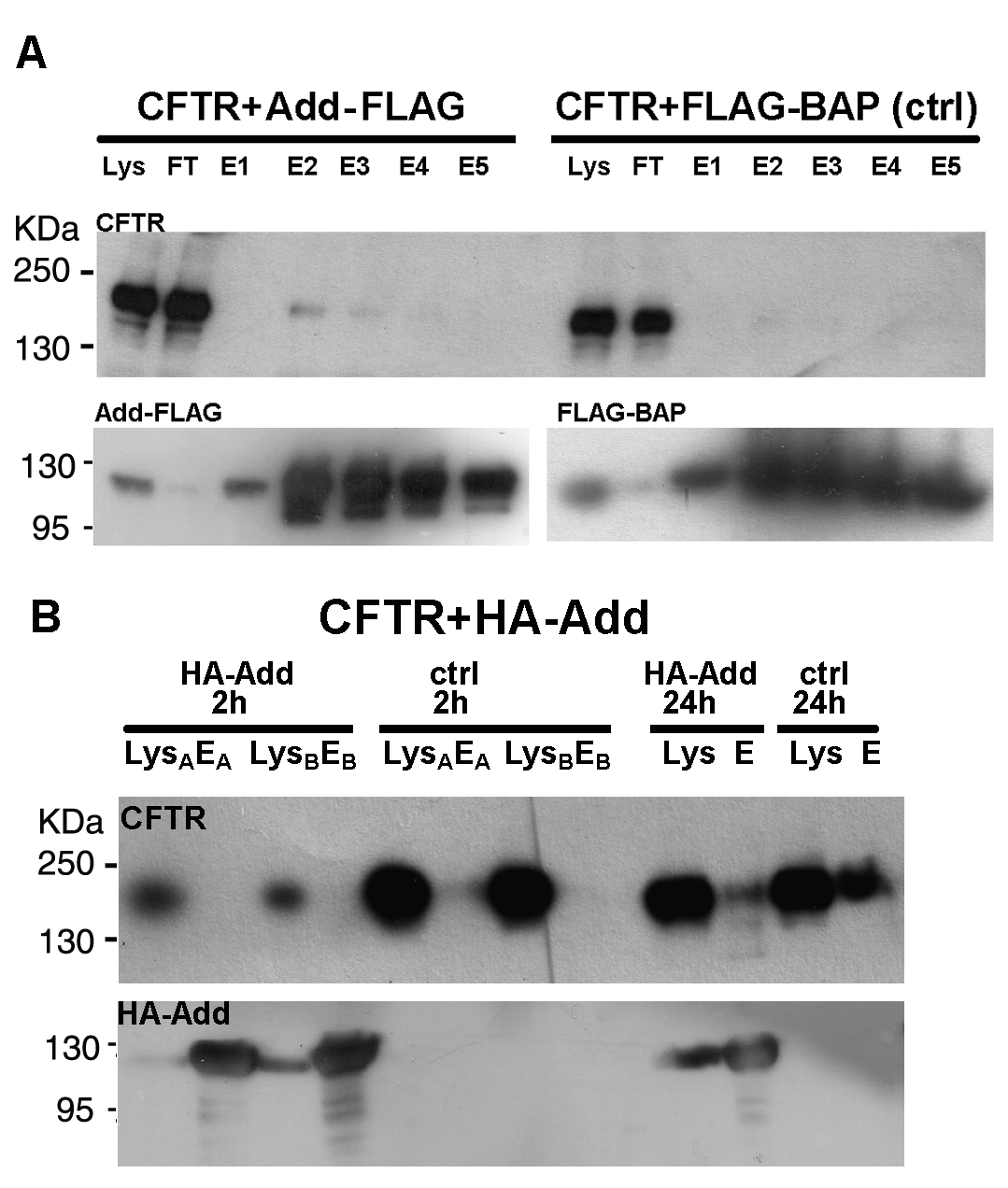

Supplement: Figure S4 — Interaction between adducin and CFTR: immunoprecipitation experiments. A) Immunoprecipitation experiment on HEK cells cotransfected with pcDNA3-CFTR and pcDNA3.1-WT adducin-FLAG (C-terminal FLAG tag, Add-FLAG); pcDNA3-CFTR and pFLAG-CMV4-BAP (bovine alkaline peroxidase, FLAG-BAP) cotransfection served as control. CFTR signal (upper panel; anti-CFTR antibody) and FLAG signal (lower panel; anti-FLAG antibody) in cell lysates (Lys), flow through (FT) and five sequential 40 µl eluates (E1–E5) are shown. B) Immunoprecipitation experiments on HEK cells cotransfected with pcDNA3-CFTR and pcDNA3.1-HA-WT adducin (N-terminal HA-tag, HA-Add). pcDNA3-CFTR and empty pFLAG-CMV4 plasmid cotransfection served as control (ctrl). CFTR signal (upper panel; anti-CFTR antibody) and HA signal (lower panel; anti-HA antibody) in cell lysates (Lys) and eluates (E) from 2 and 24 hours incubation with anti-HA agarose affinity gel are shown. Subscripts A and B refer to two independent preparations. (TIF) [file pone.0052014.s004.tif]

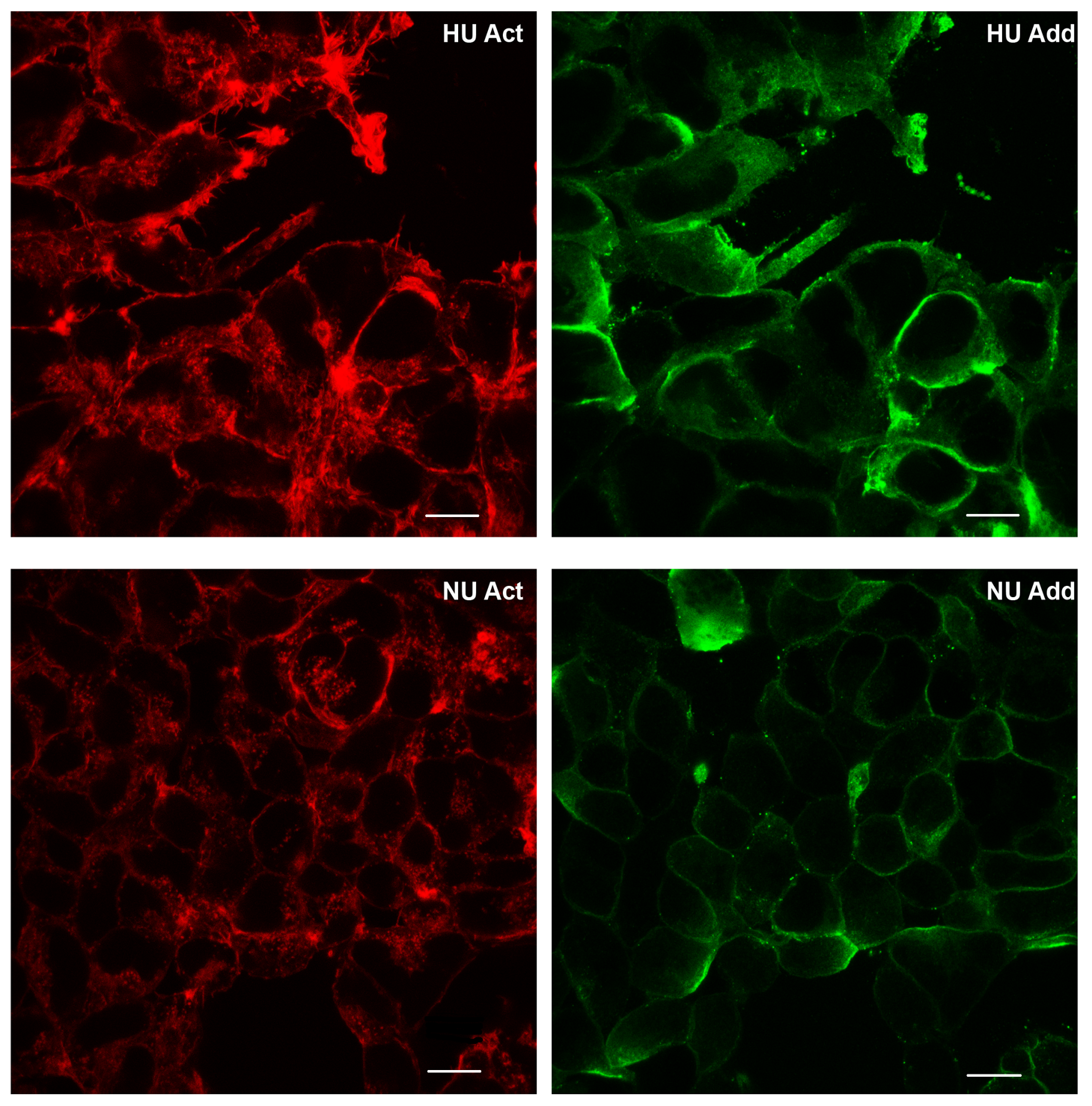

Supplement: Figure S5 — Actin and adducin staining in NU12 and HU33 cells. Confocal images showing actin (Act, left) and adducin (Add, right) in HU33 (HU, upper panels) and NU12 (NU, lower panels) cells. Primary antibody: anti-HA; secondary antibody: Alexa 488 anti-mouse. Actin fibers were stained with Alexa 568 Phalloidin. Scale bar is 10 µm. (TIF) [file pone.0052014.s005.tif]
