# Supplementary material for: Hypertension-Linked Mutation of α-Adducin Increases CFTR Surface Expression and Activity in HEK and Cultured Rat Distal Convoluted Tubule Cells
Source: PLoS One. 2012 Dec 21;7(12):e52014. doi: 10.1371/journal.pone.0052014 (PMC3528715; doi:10.1371/journal.pone.0052014)
Supplement: Text S1 — Supplementary methods. Additional methodological information concerning the results presented in the supplementary figures, i.e. evaluation of transfection efficiency, fluorescence resonance energy transfer (FRET) experiments, immunoprecipitation, HA-adducin and actin staining of HU33/NU12 cells. (DOC) [file pone.0052014.s006.doc]

**Supplementary methods**

**Evaluation of transfection efficiency**

CFTR transfection efficiency in HU33/NU12 HEK cells has been verified by immunofluorescence and by the luciferase reporter system.

*Immunofluorescence experiments*

Cells were grown on glass coverslips, fixed with 3% paraformaldehyde (for 15 minutes at room temperature) and then permeabilized by a Triton X-100 solution (0.1% Triton X-100, 3 mM MgCl_2_ in PBS). Non-specific binding was blocked with a BSA solution (5% goat serum, 0.1% BSA in PBS). Labelling of CFTR was performed with anti-CFTR (clone 24-1, R&D Systems) primary antibody (1:25 dilution in 0.5% BSA PBS solution overnight incubation), followed by anti-mouse-Alexa 568 (Invitrogen) secondary antibody (1:100 dilution in 0.5 % BSA PBS solution; for 1hour at room temperature). Images were acquired with a confocal microscope Leica TCS SP5 AOBS (Leica Microsystem, Heidelberg, Germany) provided with UV FW Lens 63x objective. All images were acquired with the same acquisition parameters.

*Luciferase assay*

The Renilla luciferase reporter assay (Promega) was used to monitor transfection efficiencies. NU12 and HU33 cells were seeded on 24 multiwell plates (2.2x10^5^ cells/well) and after 24 hours were cotransfected with 1 µg of pcDNA3-CFTR and 75 ng of pRL-TK (containing the Renilla luciferase reporter gene under the control of thymidine kinase promoter) plasmids, with the PEI method. 48 hours after transfection each well was washed with PBS and the cells were enzymatically (trypsin-EDTA) detached. 50 ul were used for cell counting with an haemocytometer (Bürker chamber) and the residual was pelleted, resuspended in the lyses buffer provided in the Luciferase assay kit and used for the measurement of protein concentration (Bradford assay) and for the Luciferase assay, according to the kit provided instructions.

Results are reported in supplementary figure S2.

**Fluorescence resonance energy transfer (FRET) experiments**

FRET experiments were performed with the acceptor photobleaching method, as reported [1]. HEK293 T cells were transfected with the plasmids pECFP-C1-add (the pECFP-C1 in which the adducin cDNA was cloned) and pEYFP-C1-CFTR (the pEYFP-C1 in which the CFTR cDNA was cloned) or with pECFP-C1 (Clontech) and pEYFP-C1-CFTR for control experiments. The FRETeff calculations were performed in a region of interest including a single cell, by using the ImageJ FRETcalc Plug-In [2] without thresholding and filtering the CFP images before the calculations. The FRETeff images were obtained by the output images of the FRETcalc plug-in, imposing a threshold of 20 grey levels for both the CFP and the YFP images.

**Immunoprecipitation**

*FLAG-adducin*

HEK cells, cotransfected with pcDNA3-CFTR and pcDNA3.1 adducin-FLAG were lysed in Buffer B (25mM Tris pH 8, 150 mM NaCl, 10% glycerol, 0.5% Triton X-100, Complete EDTA-free Protease Inhibitor Cocktail (Roche). After repetitively syringing through 20 gauge needle, cell debris were pelleted at 4500g for 10 min (4°C) and supernatants (5 mg) samples were immunoprecipitated using the anti-FLAG M2 Affinity Gel (100 µl), a purified murine IgG1monoclonal anti-FLAG antibody covalently attached to agarose beads (Sigma). The bound protein complexes were eluted in presence of enriched FLAG peptide (130 µg/ml) in PBS buffer in 40 µl aliquots and then run on SDS-PAGE and revealed by Western blotting using anti-CFTR (clone 24-1, R&D Systems) and anti-FLAG antibodies (Sigma).

*HA-adducin*

HEK cells cotransfected with pcDNA3-CFTR and pcDNA 3.1-HA-adducin WT (or empty pFLAG in the case of controls) were lysed in a Triton lyses buffer (0.1%Triton X-100, 0.1mM MES, 1mM EGTA, 0.5mm MgCl_2_, NaN_3_ 0.2 mg/ml, pH 6.5) or in buffer B (25mM Tris pH 8, 150 mM NaCl, 10% glycerol, 0.5% Triton X-100) in the presence of Complete EDTA-free Protease Inhibitor Cocktail (Roche). After repetitively syringing through 20 gauge needle, cell debris were pelleted at 4500g for 10 min (4°C) and supernatants (2 mg) were incubated with 50 µl of anti-HA agarose affinity gel (Sigma), at 4°C, for 2 h or 24 h. Bound protein complexes were eluted by resuspending the resin in 50 µl of 2X SDS-PAGE solubilising buffer and assayed by Western blotting using anti-CFTR (CFTR H-182 antibody, Santa Cruz, sc-10747) and anti-HA antibodies (Sigma).

**HA-adducin and actin staining of HU33/NU12 cells**

HU33/NU12 HEK cells were grown on glass coverslips coated with L- polylysine. The cells were washed twice with PBS and fixed with 3% paraformaldehyde (for 7 minutes at room temperature) and then permeabilized by a Triton X-100 solution (0.1% Triton X-100, 3 mM MgCl_2_ in PBS). Non-specific binding was blocked with a BSA solution (5% BSA in PBS). Staining for adducin was performed with anti-HA primary antibody (1:200 dilution in BSA solution; for 1 hour at room temperature), followed by anti-mouse-Alexa488 (Invitrogen) secondary antibody (1:300 dilution in BSA solution; for 1 hour at room temperature). In the case of actin co-staining, the incubation with the secondary antibody was followed by three washes with PBS and the staining for F-actin (supplentary figure S5) was performed with Alexa 568 phalloidin (0.16 µM in a 0.1% BSA PBS solution; for 20 min at room temperature). Coverslips, mounted in 90% glycerol, 1% DABCO, were observed with a Leica TCS SPE AOBS (Leica Microsystem, Heidelberg, Germany) provided with 63x oil objective. Same acquisition parameters were used for all conditions.

## References

1. Rodighiero S, Bazzini C, Ritter M, Furst J, Botta G, et al. (2008) Fixation, mounting and sealing with nail polish of cell specimens lead to incorrect FRET measurements using acceptor photobleaching. Cell Physiol Biochem 21: 489-498.

2. Stepensky D (2007) FRETcalc plugin for calculation of FRET in non-continuous intracellular compartments. BiochemBiophysResCommun 359: 752-758.
